# Cyclo­penta­dienone triisocyanide iron complexes: general synthesis and crystal structures of tris­(2,6-di­methyl­phenyl isocyanide)(η^4^-tetra­phenyl­cyclo­penta­dienone)iron and tris­(naphthalen-2-yl iso­cyanide)(η^4^-tetra­phenyl­cyclo­penta­dienone)iron acetone hemisolvate

**DOI:** 10.1107/S205698902300498X

**Published:** 2023-06-13

**Authors:** André Bütikofer, Peter Chen

**Affiliations:** a Department of Chemistry and Applied Biosciences, Laboratory of Organic Chemistry, ETH Zurich, Zurich 8093, Switzerland; Tulane University, USA

**Keywords:** crystal structure, isocyanide, cyclo­penta­dienone iron complex, LEDs

## Abstract

Irradiation of a toluene solution containing cyclo­penta­dienone tricarbonyl iron complexes and isocyanides with blue LEDs afforded the formation and isolation of 12 triisocyanide complexes, two of which, namely tris­(2,6-di­methyl­phenyl isocyanide)(η^4^-tetra­phenyl­cyclo­penatedienone)iron, [Fe(C_9_H_9_N)_3_(C_29_H_20_O)], and tris­(naphthalen-2-yl isocyanide)(η^4^-tetra­phenyl­cyclo­penatedienone)iron acetone hemisolvate, [Fe(C_11_H_7_N)_3_(C_29_H_20_O)]_2_·C_3_H_6_O, could be characterized crystallographically. The air-stable compounds were purified by column chroma­tography and were characterized by ^1^H NMR, ^13^C NMR, elemental analysis and HRMS. NMR and XRD data indicate generally more electron-rich Fe^0^ centers compared to the corresponding tricarbonyl compounds.

## Abstract 2

Cyclo­penta­dienone triisocyanide iron complexes were isolated and fully characterized for the first time. Two of the twelve isolated complexes could be crystallographically characterized.

## 1. Chemical context

While cyclo­penta­dienone tricarbonyl iron complexes are well known species and have been established as pre-catalysts for hydrogenation and transfer hydrogenation reactions among other types and reactions (Quintard & Rodriguez, 2014 ▸; Pignataro & Gennari, 2020 ▸), the corresponding triisocyanide complexes have thus far not been described in the literature. Considering the electronic similarity between CO and CN*R* (*R* = alkyl or ar­yl) ligands (Pruchnik & Duraj, 1990 ▸), the incorporation of isocyanide ligands into the cyclo­penta­dienone iron complex framework could open up new handles to tune the electronic and steric properties of the compounds by variation of the *R* group on the isocyanide ligands.

**Figure 1:**
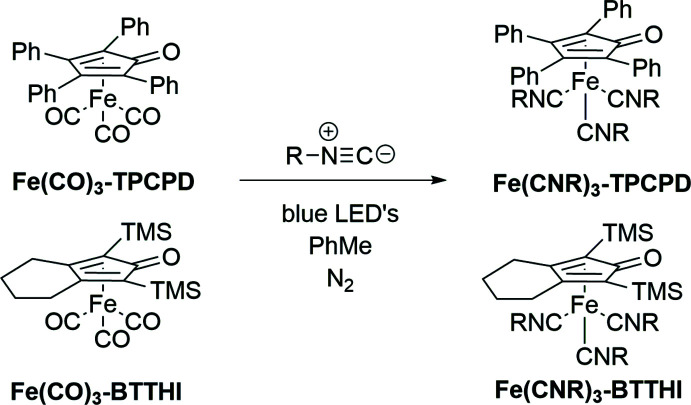
Synthetic route to access cyclo­penta­dienone triisocyanide complexes starting from the corresponding tricarbonyl complexes by irradiation with blue LEDs.

Inspired by the procedure described for obtaining cyclo­penta­dienone triaceto­nitrile complexes by irradiation of the corresponding tricarbonyl complexes with light in aceto­nitrile solution (Knölker *et al.*, 1999 ▸), it was found that irradiating a toluene solution (*ca* 0.1 *M* total concentration) of a cyclo­penta­dienone tricarbonyl complex [1 equiv., cyclo­penta­dien­one = tetra­phenyl­cyclo­penta­dienone (TPCPD) or 1,3-bis(tri­methyl­sil­yl)-4,5,6,7-tetra­hydro-2*H*-inden-2-one (BTTHI)] and a slight excess of an isocyanide *R*NC [4 equiv., *R* = CH_2_Ts (Ts = toluene­sulfon­yl), ^*t*^Bu, Bu, 2,6-DMP (DMP = di­methyl­phen­yl), 2-Naphth (Naphth = Naphth­yl), CH_2_Ph] with blue LEDs under a nitro­gen atmosphere overnight afforded the corresponding triisocyanide complexes in moderate to good yields (20–85%) (Fig. 1 ▸). For two of the twelve isolated compounds, namely **Fe(CN-2,6-DMP)_3_-TPCPD** and **Fe(CN-2-Naphth)_3_-TPCPD**, single crystals suitable for XRD could be obtained. Their structures are reported herein. 
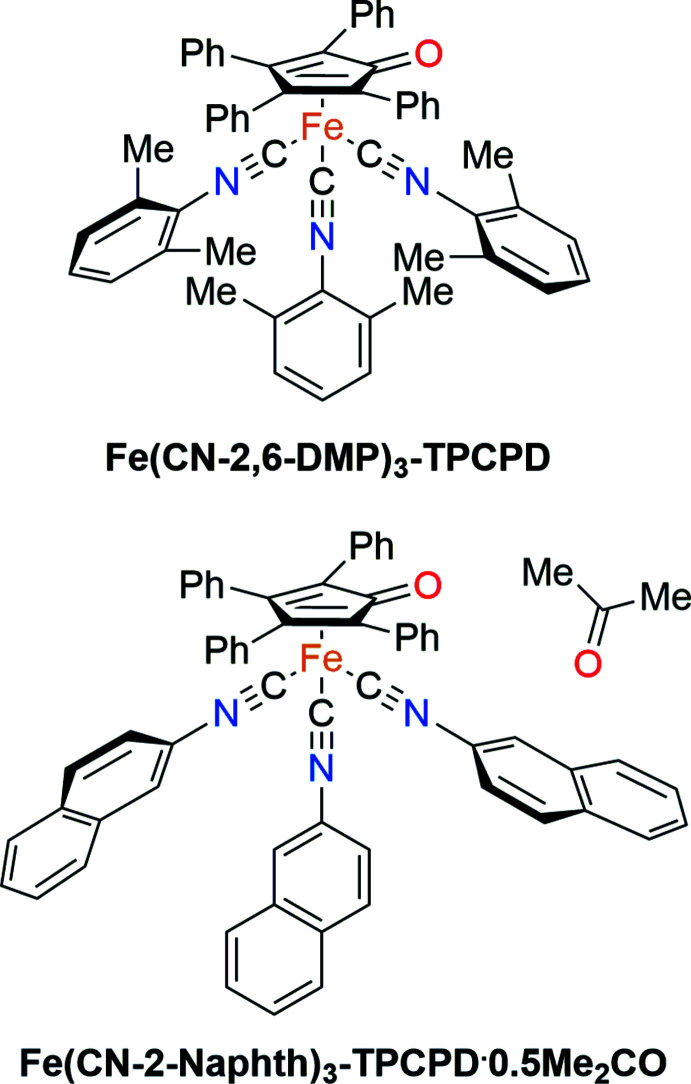


The isolated complexes were inactive in hydrogenation and transfer hydrogenation reactions of aceto­phenone in ^*i*^PrOH (1 mol% catalyst loading, 363 K, 10 bar H_2_). Addition of Me_3_NO, as is routinely done for activating the corresponding tricarbonyl complexes, did not lead to turnover either. It is assumed that neither Me_3_NO nor elevated temperatures are able to cleave one of the Fe—CN*R* bonds to free up a coord­ination site needed for catalysis. While potential applications of these complexes in catalysis were unsuccessful, our studies nevertheless prompted us to seek systematic relationships between the ligand properties and either the structural or the functional properties of the complexes.

## 2. Structural commentary

Comparisons to tricarbon­yl(η^4^-tetra­phenyl­cyclo­penta­dien­­one)iron [**Fe(CO)_3_-TPCPD**] will be based on the reported structure (Gupta *et al.*, 2000 ▸; CCDC deposition number 142285).

**Figure 2:**
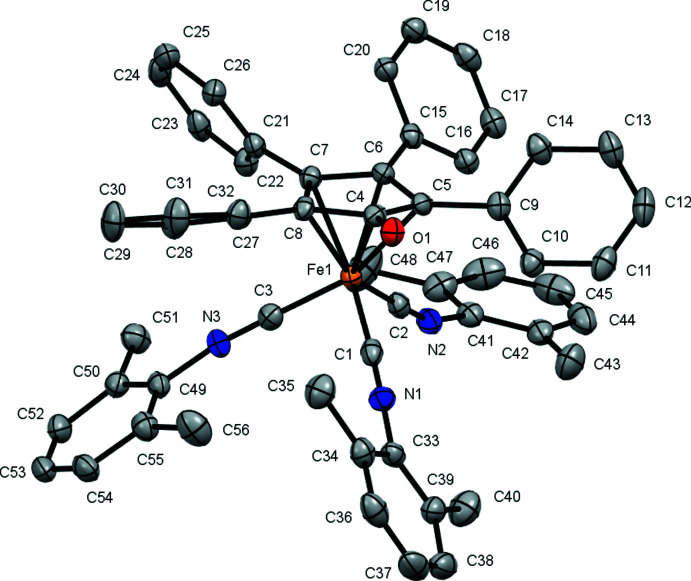
Crystal structure of **Fe(CN-2,6-DMP)_3_-TPCPD**. Displacement ellipsoids are shown at the 50% probability level. Hydrogen atoms are not shown for clarity.

**Figure 3:**
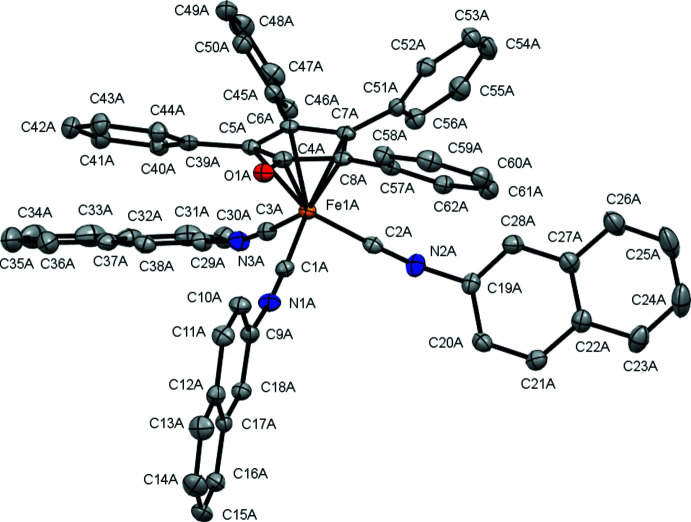
Crystal structure of **Fe(CN-2-Naphth)_3_-TPCPD**. Displacement ellipsoids are shown at the 50% probability level. The second complex in the asymmetric unit, hydrogen atoms and the co-crystallized acetone mol­ecule are not shown for clarity.

**Fe(CN-2,6-DMP)_3_-TPCPD** (Fig. 2 ▸) crystallizes in the *P*ca2_1_ space group and features one complex mol­ecule per asymmetric unit with no co-crystallized solvent mol­ecules. The average Fe—CN*R* bond distance is 1.84 (1) Å. The average C—Fe—C angle is 94 (3)°. **Fe(CN-2-Naphth)_3_-TPCPD** (Figs. 3 ▸ and 4 ▸) crystallizes in the *P*2_1_ space group and features two complex mol­ecules and a co-crystallized acetone mol­ecule in the asymmetric unit. The average Fe—CN*R* distance is 1.831 (6) Å. The average C—Fe—C angle is 96 (2)°. In one of the complex mol­ecules, there is disorder in two of the three naphthyl groups with site-occupancy factors of 0.911 (3) and 0.089 (3). **Fe(CN-2,6-DMP)_3_-TPCPD** shows a C=O double bond length of 1.243 (5) Å, while the two complexes in the crystal structure of **Fe(CN-2-Naphth)_3_-TPCPD** show lengths of 1.247 (4) and 1.243 (4) Å. These values are marginally longer than the C=O double bond in **Fe(CO)_3_-TPCPD**, in which the C=O double bond length is 1.22 (1) Å. Both compounds feature the iron atom in the formal 0 oxidation state. The cyclo­penta­dienone ligand is coordinated in an η^4^ fashion.

In **Fe(CN-2,6-DMP)_3_-TPCPD**, the average distance between Fe and the diene carbon atoms (C5, C6, C7, C8) is 2.11 (3) Å and the Fe distance to the ketonic carbon atom (C4) is 2.361 (4) Å. In **Fe(CN-2-Naphth)_3_-TPCPD**, the average distance between Fe and the diene carbon atoms (C5, C6, C7, C8) is 2.10 (3) Å and the distances from Fe to the ketonic carbon atom (C4) are 2.367 (3) and 2.373 (3) Å. Compared to values in **Fe(CO)_3_-TPCPD** [2.14 (2) Å and 2.40 (1) Å, respectively], the values measured in the iso­cyanide complexes are marginally smaller, *i.e*. the Fe–diene bond is shorter.

The envelope angles, defined as the angle between the plane spanned by C5, C6, C7 and C8 with the plane spanned by C4, C5, C8 and O1, are 14.1689 (3)° for **Fe(CN-2,6-DMP)_3_-TPCPD** and 15.6550 (2)° and 12.8805 (2)° in **Fe(CN-2-Naphth)_3_-TPCPD**. They are lower than the envelope angle for **Fe(CO)_3_-TPCPD**, which is reported to be 16°. The isocyanide complexes show a flatter cyclo­penta­dienone ligand, which, together with the elongated C=O double bonds, could indicate more ‘cyclo­penta­dienyl character’, reflecting the high electron density on iron and its presumed propensity to redistribute its electrons into the ligands.

The C—N—C angles in the isocyanide ligands in **Fe(CN-2,6-DMP)_3_-TPCPD** are 178.6 (4), 163.5 (4) and 159.5 (4)°. In **Fe(CN-2-Naphth)_3_-TPCPD**, the angles are 170.5 (3), 168.6 (3) and 168.5 (3)° for the complex without disorder and 175.8 (3), 162.6 (3) and 157.9 (12)° in the complex with disorder. The bending away from 180° indicates significant back-bonding from the Fe^0^ center into the π*_CN_ orbitals of the ligands. For **Fe(CN-2,6-DMP)_3_-TPCPD**, the isocyanide ligand showing an almost linear C—N—C angle of 178.6 (4)°, is located parallel to the C=O double bond of the cyclo­penta­dienone ligand. This could indicate that, in this position in the Fe(CN*R*)_3_ fragment, only a little back-bonding takes place, and that the Fe^0^ atom prefers to distribute its electron density into the two isocyanide ligands facing away from the C=O double bond. In **Fe(CN-2-Naphth)_3_-TPCPD**, the spread of the angles is generally lower. It can furthermore be observed that the C—N—C bends are angled towards the cyclo­penta­dienone ligand in the same mol­ecule. As the asymmetric unit features two inter­locked **Fe(CN-2-Naphth)_3_-TPCPD** mol­ecules (the C—N—C bends face away from the other complex mol­ecule in the pairs), it is proposed that the lower spread and lack of trend as to which position in the Fe(CN*R*) fragment experiences how much back-bonding is due to crystal-packing forces. This is not observed in **Fe(CN-2,6-DMP)_3_-TPCPD**, since there is no obvious inter­molecular inter­action in this case.

^13^C NMR analysis revealed similar experimental evidence for the higher electron density on iron. Table 1 ▸ shows the ^13^C NMR chemical shifts for the ring carbon atoms C4, C5, C6, C7 and C8 in the complexes **Fe(C** ***X*** **)_3_-TPCPD** (*X* = O, N*R*). The corresponding values for complexes with the BTTHI ligand are shifted *ca* 5 ppm downfield for C4 and C6/C7 and *ca* 10 ppm upfield for C5/C8 and follow the same overall trend as the complexes with TPCPD. It can be observed that the signals are all shifted upfield, *i.e.* to lower chemical shifts, compared to the parent tricarbonyl complex. This observation can be explained by considering that isocyanides are weaker π-acceptors and stronger σ-donors compared to CO. They thus render the iron center more electron rich and therefore lead to more electron density and thus shielding in the cyclo­penta­dienone ligand. Complexes with isocyanide ligands bearing electron-withdrawing or aromatic substituents (CH_2_Ts, 2-Naphth, 2,6-DMP) show more deshielded signals compared to isocyanide ligands with electron-donating substituents (CH_2_Ph, Bu, ^*t*^Bu).

Furthermore, ^13^C NMR analysis showed that the CNR signals are generally more shielded by 2–5 ppm for complexes bearing the TPCPD ligand compared to the BTTHI ligand, indicative of stronger *d*_Fe_ to π*_CN_ back-bonding with BTTHI, since more back-donation generally leads to higher chemical shifts (Pruchnik & Duraj, 1990 ▸). TPCPD can thus be said to be a stronger acceptor than BTTHI, rendering the Fe center less electron rich.

## 3. Supra­molecular features

In the crystal of **Fe(CN-2-Naphth)_3_-TPCPD**, the complexes form pairs with the Fe(CN*R*)_3_ fragments facing each other. The complexes are rotated by approximately 180° relative to each other. The naphthyl groups form an inter­locked structure. No obvious inter­molecular inter­actions are observed in **Fe(CN-2,6-DMP)_3_-TPCPD**.

## 4. Database survey

**Figure 5:**
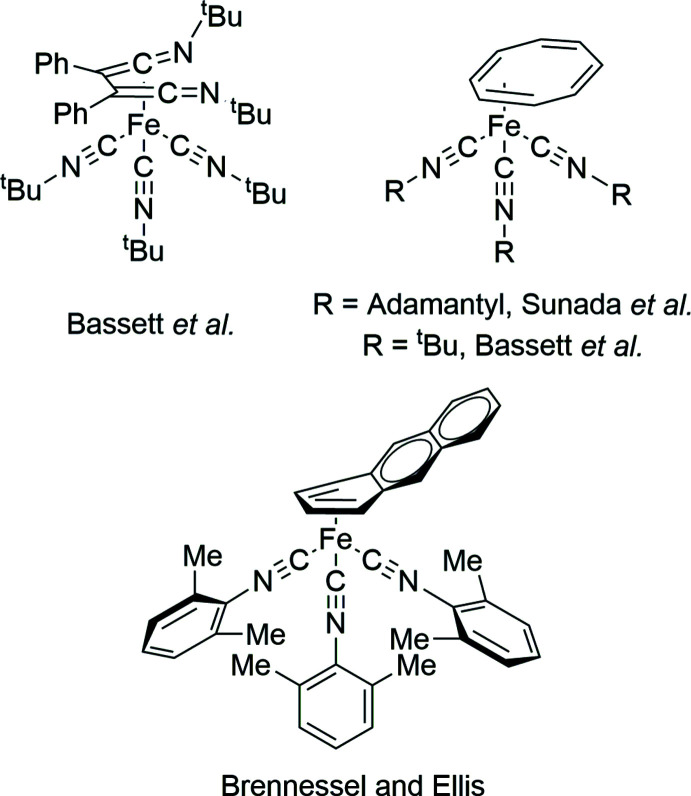
Crystallographically characterized Fe^0^ triisocyanide diene complexes reported in the CSD.

In a search of the Cambridge Structural Database (WebCSD, accessed May 2023, Groom *et al.*, 2016 ▸), three structures featuring an iron triisocyanide moiety with the iron center in the 0 oxidation state were found in which the iron is bound to a diene (Fig. 5 ▸). Bassett and co-workers reported the complex tris­(*tert*-butyl­isocyanide)(η^4^-*N* ^1^,*N* ^4^-di-*tert*-butyl-2,3-di­phenyl­buta-1,3-diene-1,4-di­imine)­iron, which could be accessed by either treating Fe_2_(CN^*t*^Bu)_9_ or Fe(CN^*t*^Bu)_5_ with di­phenyl­acetyl­ene (Bassett *et al.*, 1978 ▸, CCDC deposition number 1107207; Bassett *et al.*, 1980 ▸, CCDC deposition number 1107208). Sunada and co-workers reported the structure of tris­(η^4^-adamantyl isocyanide)(1,3,5,7-cyclo­tetra­ene)iron (Sunada *et al.*, 2015 ▸, CCDC deposition number 1416957). The compound was prepared by treating di(1,3,5,7-cyclo­tetra­ene)iron with three equivalents of adamantyl isocyanide. The analogous structure with ^*t*^BuNC instead of adamantyl iso­cyanide was also reported (Bassett *et al.*, 1981 ▸), but not crystallographically characterized. Brennessel and Ellis reported the structure of Fe(η^4^-anthracene)(CN-2,6-DMP)_3_ (Brennessel & Ellis, 2022 ▸, CCDC deposition number 2127596). The structure features the most similar coordination environment around iron compared to **Fe(CN-2,6-DMP)_3_-TPCPD** and **Fe(CN-2-Naphth)_3_-TPCPD** found in the database. The average C—Fe—C angle is 95 (5)°, which is very similar to the values observed in **Fe(CN-2,6-DMP)_3_-TPCPD** and **Fe(CN-2-Naphth)_3_-TPCPD** [94 (3)° and 96 (2)°, respectively]. The reported envelope angle between the iron coordinating η^4^-diene unit and the exo-naphthalene portion is 30.76 (16)° for Fe(η^4^-anthracene)(CN-2,6-DMP)_3_, which is significantly higher than the angle observed with the carbonyl units in the cyclo­penta­dienone complexes. This could be due to more aromatic character in the cyclo­penta­dienone ligand made possible by the ketone unit. Both tris­(η^4^-adamantylisocyanide)(1,3,5,7-cyclo­tetra­ene)iron and Fe(η^4^-anthracene)(CN-2,6-DMP)_3_ show a similar back-bonding situation to **Fe(CN-2,6-DMP)_3_-TPCPD**, namely that the isocyanide ligands on the side of the 2,3 positions of the diene (C6 and C7) show more acute C—N—C angles than the ligand on the side of the 1,4 positions (C5 and C8) [177.6 (3), 174.1 (3) and 166.5 (3)° and 170.8 (2), 162.0 (2 and 138.9 (2° for Fe(η^4^-anthracene)(CN-2,6-DMP)_3_ and tris­(η^4^-ada­man­tylisocyanide)(1,3,5,7-cyclo­tetra­ene)iron, respectively]. Since in these crystals no obvious inter­molecular inter­actions can be observed, as in the case for **Fe(CN-2,6-DMP)_3_-TPCPD**, the differences in angles depending on the position could be rationalized by electronic effects.

## 5. Synthesis and crystallization

The general procedure for the synthesis of the triisocyanide complexes is as follows: Under an atmosphere of N_2_, the iron tricarbonyl complex (1 equiv.) and the isocyanide (4 equiv.) were dissolved in toluene (*ca* 0.1 *M* total concentration). Drying or degassing of the solvent was not found to be necessary. The solution was irradiated with blue LEDs (RND Components RND 135-00259, 4.8 W, 470 nm) at room temperature overnight. The next day, the solution was directly loaded onto a silica packed column and purified by column chromatography using the appropriate eluent as indicated below. The relevant, yellow-colored fractions were combined and concentrated under reduced pressure. For complexes bearing the TPCPD ligands with electron-rich isocyanides (CNCH_2_Ph, CN^*t*^Bu, CNBu), it was necessary to perform rotary evaporation at 298 K instead of 313 K because of the thermal instability of these compounds, as evidenced by the observation of the dark-purple color of the TPCPD ligand during thin layer chromatography (TLC) analysis. The complexes were isolated as yellow to orange solids and were characterized by ^1^H NMR, ^13^C NMR, elemental analysis and HRMS. Single crystals of the compounds **Fe(CN-2,6-DMP)_3_-TPCPD** and **Fe(CN-2-Naphth)_3_-TPCPD** were obtained by suspending the solids in acetone to obtain a saturated solution, filtering off the solids and storing the saturated solution at 253 K in a freezer.

The decomposition upon heating, presumably due to the loss of the cyclo­penta­dienone ligand, appears to depend on how electron rich the complex is as a whole, as indicated by ^13^C NMR, with more electron density on the cyclo­penta­dienone ligand leading to thermal instability. Combining the observations made above, it can be said that more thermally stable complexes can be expected by combining strongly π-accepting isocyanide ligands with weakly electron-accepting cyclo­penta­dienone ligands, reminiscent of a push–pull inter­action between the cyclo­penta­dienone ligand and the isocyanide ligands mitigated by the iron center.

**Fe(CO)_3_-BTTHI** and tetra­phenyl­cyclo­penta­dienone were prepared according to literature methods (Moulin *et al.*, 2013 ▸; Liang, 2019 ▸).

**Tricarbon­yl(**η^4^-**tetra­phenyl­cyclo­penta­dienone)iron**: A dried and argon-flushed 100 ml two-necked round-bottom flask equipped with a stir bar and a reflux condenser was charged with 2.0 g tetra­phenyl­cyclo­penta­dienone (5.2 mmol, 1 equiv.), 1.88 g Fe_2_(CO)_9_ (5.2 mmol, 1 equiv.) and 50 ml dry toluene. The mixture was heated to reflux overnight. The next day, the mixture was allowed to cool to room temperature. The mixture was passed through a pad of silica and eluted first with toluene to remove Fe(CO)_5_ and unreacted tetra­phenyl­cyclo­penta­dienone. The eluent was changed to EtOAc and the orange band was collected. The orange solution was concentrated under reduced pressure. The product was obtained as a yellow–orange solid after drying *in vacuo* (2.0 g, 74%). ^1^H NMR (δ, 400 MHz, CDCl_3_): 7.64–7.54 (*m*, 4H), 7.32–7-23 (*m*, 8H), 7.23–7.14 (*m*, 8H). ^13^C NMR (δ, 100 MHz, CDCl_3_): 208.7, 170.0, 132.0, 131.0, 130.4, 130.1, 128.9, 128.21, 128.18, 128.0, 104.2, 82.7. Analysis calculated for C_32_H_20_FeO_4_ (%): C, 73.30; H, 3.84. Found: C, 73.51; H, 3.97.

**Fe(CNCH_2_Ts)_3_-BTTHI**: Yield: 54%. TLC (SiO_2_, EtOAc): *r*_f_ = 0.2. ^1^H NMR (δ, 400 MHz, CDCl_3_): 7.88 (*d*, *J* = 8.2 Hz, 6H), 7.44 (*d*, *J* = 8.2 Hz, 6H), 4.84 (*s*, 6H), 2.46 (*s*, 9H), 2.24 (*s*, *br*, 4H), 1.62 (*s*, *br*, 4H), 0.00 (*s*, 18H). ^13^C NMR (δ, 100 MHz, CDCl_3_): 188.0, 178.3, 146.5, 133.5, 130.6, 129.2, 105.5, 66.7, 64.4, 25.1, 23.0, 21.9, 0.29. HRMS (*m*/*z*): [*M* + H]^+^ calculated for C_42_H_54_FeN_3_O_7_S_3_Si_2_: 920.2006. Found: 920.1993. Analysis calculated for C_42_H_53_FeN_3_O_7_S_3_Si_2_ (%): C, 54.83; H, 5.81; N, 4.57. Found: C, 53.56; H, 5.69; N, 4.62.

**Fe(CN^t^Bu)_3_-BTTHI**: Yield: 30%. TLC (SiO_2_, EtOAc): *r*_f_ = 0.15. ^1^H NMR (δ, 400 MHz, CDCl_3_): 2.39–2.22 (*m*, 4H), 1.63 (*s*, *br*, 4H), 1.43 (*s*, 27H), 0.21 (*s*, 18H). ^13^C NMR (δ, 100 MHz, CDCl_3_): 175.0, 100.7, 62.2, 56.1, 31.6, 25.5, 23.4, 1.2 (C*O not observed*). HRMS (*m*/*z*): [*M* + H]^+^ calculated for C_30_H_54_FeN_3_OSi_2_: 584.3149. Found: 584.3165. Analysis calculated for C_30_H_53_FeN_3_OSi_2_ (%): C, 61.72; H, 9.15; N, 7.20. Found: C, 58.65; H, 8.85; N, 6.31.

**Fe(CNBu)_3_-BTTHI**: Yield: 45%. TLC (SiO_2_, EtOAc): *r*_f_ = 0.2. ^1^H NMR (δ, 300 MHz, CDCl_3_): 3.59 (*t*, *J* = 6.6 Hz, 6H), 2.40–2.23 (*m*, 4H), 1.72–1.58 [*m*, 10H, *overlapping signals from CNBu C*H_2_ *(1×) and BTTHI C*H_2_ *(2×)*], 1.57–1.38 (*m*, 6H), 0.95 (*t*, *J* = 7.3 Hz, 9H), 0.18 (*s*, 18H). ^13^C NMR (δ, 100 MHz, CDCl_3_): 174.4, 101.0, 61.6, 44.5, 32.3, 24.6, 23.4, 19.8, 13.5, 0.8. (C*O not observed).* HRMS (*m*/*z*): [*M* + H]^+^ calculated for C_30_H_54_FeN_3_OSi_2_: 584.3149. Found: 584.3136. Analysis calculated for C_30_H_53_FeN_3_OSi_2_ (%): C, 61.72; H, 9.15; N, 7.20. Found: C, 60.90; H, 8.99; N 6.77.

**Fe(CN-2,6-DMP)_3_-BTTHI**: Yield: 60%. TLC (SiO_2_, 4:1 hexa­ne/EtOAc): *r*_f_ = 0.2. ^1^H NMR (δ, 500 MHz, CDCl_3_): 7.04 (*s*, *br*, 9H), 2.71–2.63 (*m*, 2H), 2.50–2.46 (*m*, 2H), 2.44 (*s*, 18H), 1.93–1.84 (*m*, 2H), 1.84–1.74 (*m*, 2H), 0.23 (*s*, 18H). ^13^C NMR (δ, 125 MHz, CDCl_3_): 134.6, 129.9, 128.0, 126.7, 104.3, 65.7, 25.7, 23.4, 19.3, 0.9 (C*NR and* C*O not observed*). HRMS (*m*/*z*): [*M* + H]^+^ calculated for C_42_H_54_FeN_3_OSi_2_: 728.3149. Found: 728.3169. Analysis calculated for C_42_H_54_FeN_3_OSi_2_ (%): C, 69.30; H, 7.34; N, 5.77. Found: C, 68.61; H, 7.50; N, 5.65.

**Fe(CN-2-Naphth)_3_-BTTHI**: Yield: 48%. TLC (SiO_2_, 2:1 hexa­ne/EtOAc): *r*_f_ = 0.3. ^1^H NMR (δ, 400 MHz, CDCl_3_): 7.90–7.76 (*m*, 12H), 7.59–7.45 (*m*, 9H), 2.79–2.65 (*m*, 2H), 2.59–2.49 (*m*, 2H), 1.96–1.79 (*m*, 4H), 0.29 (*s*, 18H). ^13^C NMR (δ, 100 MHz, CDCl_3_): 182.8, 178.2, 133.4, 132.0, 129.7, 128.0, 127.9, 127.8, 127.3, 126.7, 124.6, 124.0, 104.8, 66.6, 25.7, 23.5, 1.0. HRMS (*m*/*z*): [*M* + H]^+^ calculated for C_48_H_48_FeN_3_OSi_2_: 794.2680. Found: 794.2669. Analysis calculated for C_48_H_47_FeN_3_OSi_2_ (%): C, 72.62; H, 5.97; N, 5.29. Found: C, 72.45; H, 6.17; N, 5.14.

**Fe(CNCH_2_Ph)_3_-BTTHI**: Yield: 72%. TLC (SiO_2_, EtOAc): *r*_f_ = 0.25. ^1^H NMR (δ, 400 MHz, CDCl_3_): 7.40–7.27 (*m*, 15H), 4.85 (*s*, 6H), 2.31–2.23 (*m*, 4H), 1.61–1.52 (*m*, 4H), 0.17 (*s*, 18H). ^13^C NMR (δ, 100 MHz, CDCl_3_): 134.6, 129.0, 128.2, 127.0, 102.3, 63.9, 48.8, 25.3, 23.2, 0.8 (C*NR and* C*O not observed*). HRMS (*m*/*z*): [*M* + H]^+^ calculated for C_39_H_48_FeN_3_OSi_2_: 686.2680. Found: 686.2669. Analysis calculated for C_39_H_47_FeN_3_OSi_2_ (%): C, 68.20; H, 7.04; N, 6.12. Found: C, 67.42; H, 7.08; N, 6.16.

**Fe(CNCH_2_Ts)_3_-TPCPD**: Yield: 50%. TLC (SiO_2_, EtOAc): *r*_f_ = 0.35. ^1^H NMR (δ, 400 MHz, CDCl_3_): 7.78 (*d*, *J* = 8.0 Hz, 6H), 7.45–7.38 (*m*, 4H), 7.32 (*d*, *J* = 8.0 Hz, 4H), 7.13–7.02 (*m*, 8H), 7.02–6.92 (*m*, 8H), 4.75 (*s*, 6H), 2.43 (*s*, 9H). ^13^C NMR (δ, 100 MHz, CDCl_3_): 184.8, 146.4, 134.4, 133.6, 133.3, 132.4, 130.9, 130.6, 129.1, 127.5, 127.45, 127.2, 126.1, 99.1, 79.5, 64.5, 21.9. (C*O not observed*). HRMS (*m*/*z*): [*M* + H]^+^ calculated for C_56_H_48_FeN_3_O_7_S_3_: 1026.1998. Found: 1026.1989. Analysis calculated for C_56_H_47_FeN_3_O_7_S_3_ (%): C, 65.55; H, 4.62; N, 4.10. Found: C, 64.41; H, 4.76; N, 4.05.

**Fe(CN^t^Bu)_3_-TPCPD**: Yield: 20%. TLC (SiO_2_, EtOAc): *r*_f_ = 0.2. ^1^H NMR (δ, 400 MHz, CDCl_3_): 7.63–7.56 (*m*, 4H), 7.21–7.16 (*m*, 4H), 7.13–6.97 (*m*, 12H), 1.28 (*s*, 27H). ^13^C NMR (δ, 100 MHz, CDCl_3_): 170.0, 161.8, 136.7, 135.5, 132.8, 131.0, 126.9, 126.7, 126.2, 124.8, 95.6, 75.0, 56.7, 31.1. HRMS (*m*/*z*): [*M* + H]^+^ calculated for C_44_H_48_FeN_3_O: 690.3141. Found: 690.3134. Analysis calculated for C_44_H_47_FeN_3_O (%): C, 76.62; H, 6.87; N, 6.09. Found: C, 74.18; H, 6.81; N, 5.80.

**Fe(CNBu)_3_-TPCPD**: Yield: 84%. TLC (SiO_2_, EtOAc): *r*_f_ = 0.2. ^1^H NMR (δ, 400 MHz, CDCl_3_): 7.65–7.58 (*m*, 4H), 7.21–7.15 (*m*, 4H), 7.12–7.00 (*m*, 12H), 3.50 (*t*, *J* = 6.6 Hz, 6H), 1.52–1.43 (*m*, 6H), 1.34–1.20 (*m*, 6H), 0.84 (*t*, *J* = 7.4 Hz, 9H). ^13^C NMR (δ, 100 MHz, CDCl_3_): 171.7, 163.3, 136.8, 135.5, 132.7, 130.6, 127.0, 126.9, 126.3, 124.8, 96.1, 74.8, 44.8, 32.0, 19.7, 13.5. HRMS (*m*/*z*): [*M* + H]^+^ calculated for C_44_H_48_FeN_3_O: 690.3141. Found: 690.3126. Analysis calculated for C_44_H_47_FeN_3_O (%): C, 76.62; H, 6.87; N, 6.09. Found: C, 75.85; H, 7.07; N, 6.00.

**Fe(CN-2,6-DMP)_3_-TPCPD**: Yield: 85%. TLC (SiO_2_, 2:1 hexa­ne/EtOAc): *r*_f_: 0.25. ^1^H NMR (δ, 500 MHz, CDCl_3_): 7.79–7.74 (*m*, 4H), 7.34–7.28 (*m*, 4H), 7.12–7.04 (*m*, 8H), 7.04–7.00 (7H), 7.00–6.95 (*m*, 6H), 2.19 (*s*, 18H). ^13^C NMR (δ, 125 MHz, CDCl_3_): 181.9, 165.9, 135.8, 134.9, 134.6, 132.8, 130.9, 129.7, 127.8, 127.3, 127.2, 127.1, 126.8, 125.5, 98.8, 77.4, 18.9. HRMS (*m*/*z*): [*M* + H]^+^ calculated for C_56_H_48_FeN_3_O: 834.3141. Found: 834.3137. Analysis calculated for C_56_H_47_FeN_3_O (%): C, 80.66; H, 5.68; N, 5.04. Found: C, 79.94; H, 5.87; N, 4.96.

**Fe(CN-2-Naphth)_3_-TPCPD**: Yield: 79%. TLC (SiO_2_, 1:1 hexa­ne/EtOAc): *r*_f_ = 0.2. ^1^H NMR (δ, 500 MHz, CDCl_3_): 7.87–7.83 (*m*, 4H), 7.83–7.79 (*m*, 3H), 7.75 (*d*, *J* = 8.8 Hz, 3H), 7.73–7.70 (*m*, 3H), 7.55–7.48 (*m*, 9H), 7.44–7.39 (*m*, 4H), 7.22–7.13 (*m*, 11H), 7.12–7.07 (*m*, 4H). ^13^C NMR (δ, 125 MHz, CDCl_3_): 180.2, 165.3, 135.6, 134.5, 133.2, 132.8, 132.1, 131.1, 129.4, 127.9, 127.7, 127.6, 127.49, 127.46, 127.2, 127.0, 126.8, 125.8, 124.6, 123.8, 98.8, 78.5. HRMS (*m*/*z*): [*M* + H]^+^ calculated for C_62_H_42_FeN_3_O: 900.2672. Found: 900.2649. Analysis calculated for C_62_H_41_FeN_3_O (%): C, 82.75; H, 4.59; N, 4.67. Found: C, 79.73; H, 4.82; N, 4.36.

**Fe(CNCH_2_Ph)_3_-TPCPD**: Yield: 48%. TLC (SiO_2_, EtOAc): *r*_f_ = 0.2. ^1^H NMR (δ, 300 MHz, CDCl_3_): 7.65–7.57 (*m*, 4H), 7.25–7.11 (*m*, 12H), 7.11–6.98 (*m*, 15H), 6.99–6.88 (*m*, 4H), 4.72 (*s*, 6H). ^13^C NMR (δ, 100 MHz, CDCl_3_): 173.6, 163.6, 136.2, 134.9, 133.9, 132.7, 130.9, 128.9, 128.0, 127.1, 127.03, 126.96, 126.4, 125.2, 96.6, 76.0, 48.9. HRMS (*m*/*z*): [*M* + H]^+^ calculated for C_53_H_42_FeN_3_O: 792.2672. Found: 792.2672. Analysis calculated for C_53_H_41_FeN_3_O (%): C, 80.40; H, 5.22; N, 5.31. Found: C, 77.91; H, 5.26; N, 5.12.

## 6. Refinement

Crystal data, data collection and structure refinement details are summarized in Table 2 ▸. H atoms were positioned geometrically (C—H = 0.95–0.98 Å) and refined as riding with *U*_iso_(H) = 1.2–1.5*U*_eq_(H). The crystal for **Fe(CN-2-Naphth)_3_-TPCPD** was twinned. Two domains, with approximate refined mass fractions of 3:1 and rotated by approximately 179°, were found and integrated simultaneously. The best model in terms of residual densities and their location, *R* values and weighting scheme was obtained using de-twinned HKLF4 data.

## Supplementary Material

Crystal structure: contains datablock(s) I, twin. DOI: 10.1107/S205698902300498X/mw2197sup1.cif

Structure factors: contains datablock(s) I. DOI: 10.1107/S205698902300498X/mw2197Isup2.hkl

Structure factors: contains datablock(s) twin. DOI: 10.1107/S205698902300498X/mw2197twinsup3.hkl

CCDC references: 2248733, 2260413

Additional supporting information:  crystallographic information; 3D view; checkCIF report

## Figures and Tables

**Figure 4:**
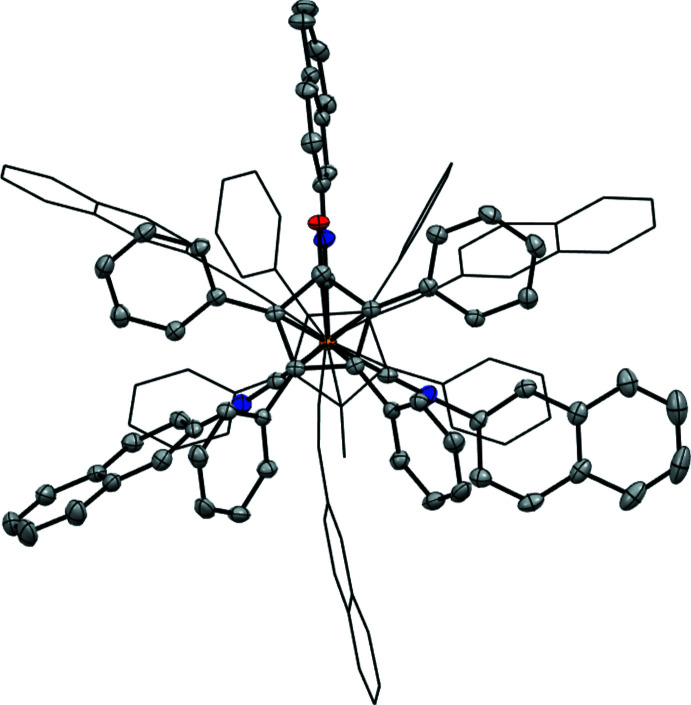
The two independent **Fe(CN-2-Naphth)_3_-TPCPD** mol­ecules in the asymmetric unit viewed along the Fe–Fe axis. In the crystal, the mol­ecules appear in pairs that are rotated by 180° with respect to each other and show an inter­locked arrangement of the naphthyl groups.

**Table 1:** ^13^C NMR chemical shifts (in p.p.m.) for the cyclo­penta­dienone ring carbon atoms (C4, C5, C6, C7, C8) (TPCPD / BTTHI)

*X*	C4	C5/C8	C6/C7
O	170.0 / 181.4	82.7 / 71.9	104.2 / 111.1
NCH_2_Ts	n/a / 178.3	79.5 / 66.7	99.1 / 105.5
N-2-Naphth	165.3 / 178.2	78.5 / 66.6	98.8 / 104.8
N-2,6-DMP	165.9 / n/a	77.4 / 65.7	98.8 / 104.3
NCH_2_Ph	163.6 / n/a	76.0 / 63.9	96.6 / 102.3
NBu	163.3 / n/a	74.8 / 61.6	96.1 / 101.0
N^*t*^Bu	161.8 / 175.0	75.1 / 62.2	95.6 / 100.7

All spectra recorded in CDCl_3_. Spectra were referenced to the solvent signal at 77.16 p.p.m. (Fulmer *et al.*, 2010 ▸). ^13^C NMR data for **Fe(CO)_3_-BTTHI** were obtained from the literature (Moulin *et al.*, 2013 ▸).

**Table 2:** Experimental details

	**Fe(CN-2,6-DMP)_3_-TPCPD**	**Fe(CN-2-Naphth)_3_-TPCPD**
Crystal data		
Chemical formula	[Fe(C_9_H_9_N)_3_(C_29_H_20_O)]	[Fe(C_11_H_7_N)_3_(C_29_H_20_O)]_2_·C_3_H_6_O
*M*_r_	833.81	1857.73
Crystal system, space group	Orthorhombic, *P* *c* *a*2_1_	Monoclinic, *P*2_1_
Temperature (K)	100	100
*a*, *b*, *c* (Å)	16.7473 (3), 12.1998 (2), 20.9668 (4)	15.2471 (1), 19.8604 (1), 15.5785 (1)
α, β, γ (°)	90, 90, 90	90, 99.378 (1), 90
*V* (Å^3^)	4283.81 (13)	4654.33 (5)
*Z*	4	2
Radiation type	Cu *K*α	Cu *K*α
μ (mm^−1^)	3.16	2.98
Crystal size (mm)	0.14 × 0.03 × 0.02	0.18 × 0.07 × 0.05

Data collection		
Diffractometer	XtaLAB Synergy, Dualflex, Pilatus 300K	XtaLAB Synergy, Dualflex, Pilatus 300K
Absorption correction	Gaussian (*CrysAlis PRO*; Rigaku OD, 2021 ▸)	Gaussian (*CrysAlis PRO*; Rigaku OD, 2021 ▸)
*T*_min_, *T*_max_	0.697, 1.000	0.779, 1.000
No. of measured, independent and observed [*I* > 2σ(*I*)] reflections	33622, 7440, 6449	89473, 18788, 17761
*R*_int_	0.081	0.050
(sin θ/λ)_max_ (Å^−1^)	0.639	0.636

Refinement		
*R*[*F* ^2^ > 2σ(*F* ^2^)], *wR*(*F* ^2^), *S*	0.042, 0.109, 1.05	0.036, 0.100, 1.07
No. of reflections	7440	18788
No. of parameters	556	1390
No. of restraints	1	1606
H-atom treatment	H-atom parameters constrained	H-atom parameters constrained
Δρ_max_, Δρ_min_ (e Å^−3^)	0.36, −0.40	0.28, −0.37
Absolute structure	Flack *x* determined using 1989 quotients [(*I* ^+^)-(*I* ^−^)]/[(*I* ^+^)+(*I* ^−^)] (Parsons *et al.*, 2013 ▸)	Classical Flack (1983 ▸) method preferred over Parsons because s.u. lower
Absolute structure parameter	−0.008 (4)	−0.004 (2)

Computer programs: *CrysAlis PRO* (Rigaku OD, 2021 ▸, 2022 ▸), *SHELXT* (Sheldrick, 2015*a* ▸), *SHELXL2018/3* (Sheldrick, 2015*b* ▸), *OLEX2* (Dolomanov *et al.*, 2009 ▸) and *Mercury* (Macrae *et al.*, 2020 ▸).

## supplementary crystallographic information

### Tris(2,6-dimethylphenyl isocyanide)(η4-tetraphenylcyclopentadienone)iron (I) Crystal data

**Table:**

[Fe(C_9_H_9_N)_3_(C_29_H_20_O)]	*D*_x_ = 1.293 Mg m^−^^3^
*M**_r_* = 833.81	Cu *K*α radiation, λ = 1.54184 Å
Orthorhombic, *P**c**a*2_1_	Cell parameters from 11706 reflections
*a* = 16.7473 (3) Å	θ = 3.6–79.3°
*b* = 12.1998 (2) Å	µ = 3.16 mm^−^^1^
*c* = 20.9668 (4) Å	*T* = 100 K
*V* = 4283.81 (13) Å^3^	Needle, clear yellow
*Z* = 4	0.14 × 0.03 × 0.02 mm
*F*(000) = 1752	

### Tris(2,6-dimethylphenyl isocyanide)(η4-tetraphenylcyclopentadienone)iron (I) Data collection

**Table:**

XtaLAB Synergy, Dualflex, Pilatus 300K diffractometer	7440 independent reflections
Radiation source: micro-focus sealed X-ray tube, PhotonJet (Cu) X-ray Source	6449 reflections with *I* > 2σ(*I*)
Mirror monochromator	*R*_int_ = 0.081
Detector resolution: 5.8140 pixels mm^-1^	θ_max_ = 80.1°, θ_min_ = 3.6°
ω scans	*h* = −21→20
Absorption correction: gaussian (CrysAlisPro; Rigaku OD, 2021)	*k* = −15→15
*T*_min_ = 0.697, *T*_max_ = 1.000	*l* = −26→21
33622 measured reflections	

### Tris(2,6-dimethylphenyl isocyanide)(η4-tetraphenylcyclopentadienone)iron (I) Refinement

**Table:**

Refinement on *F*^2^	Hydrogen site location: inferred from neighbouring sites
Least-squares matrix: full	H-atom parameters constrained
*R*[*F*^2^ > 2σ(*F*^2^)] = 0.042	*w* = 1/[σ^2^(*F*_o_^2^) + (0.063*P*)^2^] where *P* = (*F*_o_^2^ + 2*F*_c_^2^)/3
*wR*(*F*^2^) = 0.109	(Δ/σ)_max_ < 0.001
*S* = 1.05	Δρ_max_ = 0.36 e Å^−^^3^
7440 reflections	Δρ_min_ = −0.40 e Å^−^^3^
556 parameters	Absolute structure: Flack *x* determined using 1989 quotients [(*I*^+^)-(*I*^-^)]/[(*I*^+^)+(*I*^-^)] (Parsons *et al.*, 2013)
1 restraint	Absolute structure parameter: −0.008 (4)
Primary atom site location: dual	

### Tris(2,6-dimethylphenyl isocyanide)(η4-tetraphenylcyclopentadienone)iron (I) Special details

**Table:**

Geometry. All esds (except the esd in the dihedral angle between two l.s. planes) are estimated using the full covariance matrix. The cell esds are taken into account individually in the estimation of esds in distances, angles and torsion angles; correlations between esds in cell parameters are only used when they are defined by crystal symmetry. An approximate (isotropic) treatment of cell esds is used for estimating esds involving l.s. planes.

### Tris(2,6-dimethylphenyl isocyanide)(η4-tetraphenylcyclopentadienone)iron (I) Fractional atomic coordinates and isotropic or equivalent isotropic displacement parameters (Å^2^)

**Table:**

	*x*	*y*	*z*	*U*_iso_*/*U*_eq_	
Fe1	0.44212 (3)	0.29727 (4)	0.49025 (3)	0.01969 (13)	
O1	0.34098 (16)	0.1785 (2)	0.37047 (13)	0.0247 (6)	
N1	0.5466 (2)	0.2208 (3)	0.38217 (18)	0.0258 (7)	
N2	0.5401 (2)	0.2204 (3)	0.60128 (19)	0.0278 (7)	
N3	0.51844 (17)	0.5183 (2)	0.48677 (19)	0.0255 (6)	
C1	0.5072 (2)	0.2484 (3)	0.4249 (2)	0.0259 (9)	
C2	0.5031 (2)	0.2504 (3)	0.55733 (19)	0.0241 (8)	
C3	0.4886 (2)	0.4325 (3)	0.4898 (2)	0.0240 (7)	
C4	0.3418 (2)	0.2250 (3)	0.4232 (2)	0.0219 (8)	
C5	0.35340 (18)	0.1724 (2)	0.4859 (2)	0.0210 (6)	
C6	0.3355 (2)	0.2502 (3)	0.53539 (19)	0.0199 (7)	
C7	0.3282 (2)	0.3565 (3)	0.50543 (18)	0.0208 (7)	
C8	0.3381 (2)	0.3442 (3)	0.43788 (18)	0.0211 (7)	
C9	0.3600 (2)	0.0516 (2)	0.4933 (2)	0.0233 (7)	
C10	0.4154 (2)	−0.0087 (3)	0.4587 (2)	0.0270 (8)	
H10	0.451113	0.028206	0.430856	0.032*	
C11	0.4194 (3)	−0.1221 (3)	0.4642 (2)	0.0337 (9)	
H11	0.457570	−0.161958	0.439927	0.040*	
C12	0.3681 (3)	−0.1774 (3)	0.5049 (2)	0.0356 (10)	
H12	0.371492	−0.254748	0.509268	0.043*	
C13	0.3116 (3)	−0.1186 (3)	0.5392 (2)	0.0334 (9)	
H13	0.275669	−0.155950	0.566711	0.040*	
C14	0.3076 (3)	−0.0055 (3)	0.5335 (2)	0.0297 (8)	
H14	0.268673	0.033951	0.557136	0.036*	
C15	0.3175 (2)	0.2258 (3)	0.6031 (2)	0.0224 (7)	
C16	0.3635 (2)	0.1555 (3)	0.64050 (19)	0.0250 (8)	
H16	0.410074	0.122967	0.622858	0.030*	
C17	0.3424 (2)	0.1321 (3)	0.7032 (2)	0.0289 (8)	
H17	0.375556	0.086079	0.728307	0.035*	
C18	0.2733 (2)	0.1755 (3)	0.7291 (2)	0.0286 (8)	
H18	0.258942	0.159492	0.771960	0.034*	
C19	0.2253 (2)	0.2427 (3)	0.6918 (2)	0.0284 (8)	
H19	0.176829	0.270689	0.708802	0.034*	
C20	0.2475 (2)	0.2692 (3)	0.62986 (19)	0.0241 (8)	
H20	0.214970	0.317101	0.605363	0.029*	
C21	0.3020 (2)	0.4560 (3)	0.54132 (19)	0.0229 (7)	
C22	0.3432 (2)	0.4931 (3)	0.59502 (19)	0.0263 (8)	
H22	0.392299	0.459712	0.606579	0.032*	
C23	0.3130 (3)	0.5785 (3)	0.6317 (2)	0.0296 (8)	
H23	0.341359	0.602888	0.668332	0.036*	
C24	0.2411 (3)	0.6282 (3)	0.6147 (2)	0.0323 (9)	
H24	0.219540	0.685330	0.640286	0.039*	
C25	0.2011 (2)	0.5939 (3)	0.5604 (2)	0.0314 (9)	
H25	0.153037	0.629211	0.547934	0.038*	
C26	0.2311 (2)	0.5081 (3)	0.5241 (2)	0.0269 (8)	
H26	0.203041	0.484711	0.487102	0.032*	
C27	0.3286 (2)	0.4318 (3)	0.38982 (19)	0.0241 (8)	
C28	0.3420 (2)	0.5434 (3)	0.4046 (2)	0.0281 (8)	
H28	0.363307	0.562470	0.445112	0.034*	
C29	0.3246 (3)	0.6253 (3)	0.3611 (2)	0.0308 (9)	
H29	0.332359	0.699903	0.372456	0.037*	
C30	0.2959 (3)	0.5993 (3)	0.3011 (2)	0.0381 (10)	
H30	0.283663	0.655662	0.271401	0.046*	
C31	0.2851 (3)	0.4899 (3)	0.2846 (2)	0.0352 (9)	
H31	0.266627	0.471371	0.243156	0.042*	
C32	0.3011 (2)	0.4076 (3)	0.3285 (2)	0.0272 (8)	
H32	0.293296	0.333263	0.316540	0.033*	
C33	0.5933 (2)	0.1901 (3)	0.33020 (19)	0.0245 (8)	
C34	0.5665 (2)	0.2148 (3)	0.2688 (2)	0.0278 (9)	
C35	0.4882 (3)	0.2716 (4)	0.2592 (2)	0.0394 (10)	
H35A	0.446900	0.234730	0.284361	0.059*	
H35B	0.473743	0.269284	0.213920	0.059*	
H35C	0.492766	0.348139	0.272994	0.059*	
C36	0.6154 (3)	0.1832 (3)	0.2180 (2)	0.0363 (10)	
H36	0.598949	0.197116	0.175438	0.044*	
C37	0.6878 (3)	0.1316 (3)	0.2294 (2)	0.0406 (11)	
H37	0.721016	0.112063	0.194436	0.049*	
C38	0.7122 (3)	0.1085 (3)	0.2905 (2)	0.0370 (10)	
H38	0.761845	0.072581	0.297136	0.044*	
C39	0.6658 (2)	0.1366 (3)	0.3426 (2)	0.0298 (9)	
C40	0.6909 (3)	0.1127 (4)	0.4099 (3)	0.0440 (11)	
H40A	0.697759	0.181828	0.433163	0.066*	
H40B	0.741584	0.072510	0.409616	0.066*	
H40C	0.649900	0.068351	0.430990	0.066*	
C41	0.5568 (2)	0.1777 (3)	0.6620 (2)	0.0263 (8)	
C42	0.5805 (2)	0.0682 (3)	0.6658 (2)	0.0293 (8)	
C43	0.5935 (3)	0.0009 (3)	0.6074 (3)	0.0432 (11)	
H43A	0.541778	−0.021491	0.589824	0.065*	
H43B	0.624754	−0.064352	0.618305	0.065*	
H43C	0.622491	0.044347	0.575618	0.065*	
C44	0.5888 (3)	0.0228 (4)	0.7264 (3)	0.0397 (11)	
H44	0.604670	−0.051553	0.730785	0.048*	
C45	0.5742 (3)	0.0855 (5)	0.7802 (3)	0.0460 (12)	
H45	0.579116	0.053327	0.821313	0.055*	
C46	0.5524 (3)	0.1944 (4)	0.7748 (3)	0.0452 (12)	
H46	0.543125	0.236364	0.812217	0.054*	
C47	0.5439 (3)	0.2434 (4)	0.7156 (2)	0.0352 (10)	
C48	0.5181 (4)	0.3609 (4)	0.7074 (3)	0.0601 (16)	
H48A	0.549030	0.394599	0.672876	0.090*	
H48B	0.527450	0.401122	0.747135	0.090*	
H48C	0.461175	0.363298	0.696745	0.090*	
C49	0.5590 (2)	0.6118 (3)	0.46513 (19)	0.0251 (8)	
C50	0.5748 (2)	0.6956 (3)	0.5084 (2)	0.0272 (8)	
C51	0.5468 (3)	0.6862 (3)	0.5761 (2)	0.0335 (9)	
H51A	0.488492	0.679505	0.576812	0.050*	
H51B	0.562872	0.751732	0.599833	0.050*	
H51C	0.570728	0.621185	0.595776	0.050*	
C52	0.6185 (2)	0.7852 (3)	0.4862 (3)	0.0323 (8)	
H52	0.632142	0.842696	0.514717	0.039*	
C53	0.6423 (2)	0.7911 (3)	0.4229 (2)	0.0330 (9)	
H53	0.670757	0.853558	0.408235	0.040*	
C54	0.6251 (2)	0.7071 (3)	0.3812 (2)	0.0331 (9)	
H54	0.641741	0.712719	0.338044	0.040*	
C55	0.5837 (2)	0.6142 (3)	0.4012 (2)	0.0309 (9)	
C56	0.5695 (3)	0.5184 (4)	0.3583 (2)	0.0438 (11)	
H56A	0.596322	0.453630	0.375809	0.066*	
H56B	0.590747	0.534355	0.315812	0.066*	
H56C	0.511980	0.504244	0.355315	0.066*	

### Tris(2,6-dimethylphenyl isocyanide)(η4-tetraphenylcyclopentadienone)iron (I) Atomic displacement parameters (Å^2^)

**Table:**

	*U*^11^	*U*^22^	*U*^33^	*U*^12^	*U*^13^	*U*^23^
Fe1	0.0222 (2)	0.0168 (2)	0.0200 (3)	−0.00091 (18)	0.0003 (3)	0.0004 (2)
O1	0.0310 (14)	0.0216 (11)	0.0215 (15)	0.0005 (10)	−0.0023 (11)	−0.0012 (10)
N1	0.0261 (17)	0.0258 (15)	0.026 (2)	−0.0001 (12)	0.0045 (14)	−0.0020 (13)
N2	0.0252 (16)	0.0279 (15)	0.030 (2)	−0.0020 (13)	−0.0012 (15)	0.0070 (15)
N3	0.0288 (14)	0.0203 (12)	0.0273 (16)	−0.0042 (10)	−0.0006 (15)	0.0011 (14)
C1	0.0249 (19)	0.0188 (15)	0.034 (3)	−0.0028 (14)	−0.0081 (18)	0.0025 (15)
C2	0.0270 (19)	0.0181 (15)	0.027 (2)	−0.0033 (13)	−0.0001 (17)	−0.0010 (14)
C3	0.0262 (16)	0.0259 (15)	0.0197 (18)	0.0019 (12)	−0.0009 (17)	0.0007 (16)
C4	0.0197 (17)	0.0207 (15)	0.025 (2)	−0.0003 (13)	−0.0009 (15)	−0.0027 (14)
C5	0.0188 (14)	0.0172 (13)	0.0270 (19)	−0.0026 (10)	0.0010 (17)	−0.0038 (16)
C6	0.0177 (16)	0.0190 (14)	0.023 (2)	−0.0017 (12)	0.0010 (14)	0.0010 (14)
C7	0.0219 (16)	0.0192 (14)	0.021 (2)	0.0002 (12)	−0.0009 (13)	−0.0009 (13)
C8	0.0195 (17)	0.0179 (15)	0.026 (2)	0.0014 (12)	−0.0052 (14)	0.0002 (14)
C9	0.0290 (16)	0.0202 (14)	0.0206 (19)	−0.0010 (12)	−0.0047 (17)	−0.0004 (16)
C10	0.0296 (19)	0.0218 (16)	0.030 (2)	−0.0014 (14)	−0.0023 (16)	−0.0044 (15)
C11	0.041 (2)	0.0235 (17)	0.037 (2)	0.0063 (15)	−0.0090 (18)	−0.0079 (16)
C12	0.053 (3)	0.0182 (15)	0.035 (3)	0.0018 (16)	−0.0157 (19)	0.0020 (15)
C13	0.053 (3)	0.0231 (16)	0.024 (2)	−0.0080 (17)	−0.0036 (19)	0.0026 (16)
C14	0.039 (2)	0.0248 (17)	0.026 (2)	−0.0059 (15)	0.0000 (17)	−0.0030 (15)
C15	0.0235 (17)	0.0185 (14)	0.025 (2)	−0.0040 (12)	−0.0021 (16)	−0.0019 (14)
C16	0.0270 (18)	0.0222 (15)	0.026 (2)	−0.0022 (13)	0.0007 (15)	0.0008 (14)
C17	0.035 (2)	0.0249 (16)	0.027 (2)	−0.0061 (15)	−0.0033 (16)	0.0035 (15)
C18	0.038 (2)	0.0278 (17)	0.020 (2)	−0.0089 (15)	0.0047 (17)	−0.0019 (15)
C19	0.0304 (19)	0.0258 (17)	0.029 (2)	−0.0030 (15)	0.0060 (16)	−0.0047 (15)
C20	0.0244 (18)	0.0217 (15)	0.026 (2)	−0.0017 (13)	0.0018 (15)	0.0004 (14)
C21	0.0255 (18)	0.0170 (14)	0.026 (2)	−0.0030 (13)	0.0003 (15)	0.0006 (14)
C22	0.032 (2)	0.0230 (16)	0.024 (2)	−0.0008 (14)	0.0003 (16)	0.0015 (14)
C23	0.044 (2)	0.0198 (15)	0.025 (2)	−0.0057 (15)	0.0055 (17)	−0.0016 (15)
C24	0.046 (2)	0.0188 (15)	0.032 (2)	−0.0007 (15)	0.0160 (19)	−0.0039 (15)
C25	0.031 (2)	0.0255 (17)	0.038 (3)	0.0020 (15)	0.0082 (18)	0.0041 (16)
C26	0.0271 (18)	0.0208 (15)	0.033 (2)	0.0017 (14)	0.0005 (16)	0.0017 (15)
C27	0.0231 (18)	0.0234 (16)	0.026 (2)	−0.0006 (13)	0.0033 (15)	−0.0002 (14)
C28	0.034 (2)	0.0219 (16)	0.028 (2)	0.0001 (15)	0.0001 (17)	0.0010 (15)
C29	0.045 (2)	0.0189 (15)	0.029 (2)	0.0008 (15)	−0.0017 (18)	0.0030 (15)
C30	0.057 (3)	0.0260 (18)	0.032 (3)	0.0050 (18)	−0.005 (2)	0.0089 (16)
C31	0.052 (3)	0.0304 (18)	0.023 (2)	0.0019 (18)	−0.005 (2)	0.0032 (17)
C32	0.034 (2)	0.0227 (16)	0.025 (2)	0.0029 (14)	−0.0006 (16)	−0.0026 (15)
C33	0.0279 (19)	0.0210 (15)	0.025 (2)	−0.0031 (13)	0.0051 (16)	−0.0022 (14)
C34	0.036 (2)	0.0228 (16)	0.025 (2)	−0.0030 (15)	−0.0001 (17)	−0.0035 (14)
C35	0.043 (3)	0.039 (2)	0.036 (3)	0.0051 (19)	−0.007 (2)	0.0013 (18)
C36	0.052 (3)	0.0286 (19)	0.028 (2)	−0.0115 (18)	0.006 (2)	−0.0041 (17)
C37	0.048 (3)	0.031 (2)	0.043 (3)	−0.0051 (18)	0.022 (2)	−0.0098 (19)
C38	0.031 (2)	0.0283 (18)	0.051 (3)	−0.0012 (16)	0.009 (2)	−0.0083 (18)
C39	0.0281 (19)	0.0234 (16)	0.038 (3)	0.0002 (14)	0.0027 (17)	−0.0037 (16)
C40	0.043 (3)	0.043 (2)	0.045 (3)	0.008 (2)	−0.010 (2)	−0.002 (2)
C41	0.0236 (19)	0.0289 (17)	0.026 (2)	−0.0015 (14)	−0.0038 (16)	0.0023 (16)
C42	0.0234 (18)	0.0296 (18)	0.035 (2)	0.0001 (14)	−0.0050 (17)	0.0051 (17)
C43	0.050 (3)	0.032 (2)	0.048 (3)	0.0078 (18)	−0.003 (2)	−0.006 (2)
C44	0.033 (2)	0.037 (2)	0.049 (3)	−0.0057 (17)	−0.009 (2)	0.016 (2)
C45	0.034 (2)	0.075 (3)	0.029 (3)	−0.009 (2)	−0.0069 (19)	0.014 (3)
C46	0.038 (2)	0.069 (3)	0.029 (3)	0.003 (2)	−0.005 (2)	−0.012 (2)
C47	0.032 (2)	0.042 (2)	0.032 (3)	0.0019 (18)	−0.0067 (19)	−0.008 (2)
C48	0.068 (4)	0.050 (3)	0.062 (4)	0.021 (3)	−0.024 (3)	−0.026 (3)
C49	0.0253 (17)	0.0208 (16)	0.029 (2)	−0.0012 (13)	−0.0029 (15)	0.0063 (14)
C50	0.0263 (18)	0.0222 (16)	0.033 (2)	0.0015 (13)	−0.0022 (15)	0.0013 (15)
C51	0.035 (2)	0.0312 (19)	0.034 (3)	−0.0050 (16)	0.0024 (18)	−0.0064 (17)
C52	0.0310 (18)	0.0212 (14)	0.045 (2)	−0.0027 (12)	0.002 (2)	−0.0024 (18)
C53	0.0257 (19)	0.0243 (17)	0.049 (3)	−0.0022 (15)	0.0031 (18)	0.0094 (17)
C54	0.031 (2)	0.0333 (19)	0.034 (3)	−0.0034 (15)	0.0037 (17)	0.0109 (18)
C55	0.033 (2)	0.0288 (18)	0.031 (2)	−0.0020 (15)	−0.0021 (17)	0.0040 (16)
C56	0.057 (3)	0.045 (2)	0.029 (3)	−0.012 (2)	0.004 (2)	−0.002 (2)

### Tris(2,6-dimethylphenyl isocyanide)(η4-tetraphenylcyclopentadienone)iron (I) Geometric parameters (Å, º)

**Table:**

Fe1—C1	1.850 (4)	C28—C29	1.385 (5)
Fe1—C2	1.830 (4)	C29—H29	0.9500
Fe1—C3	1.825 (3)	C29—C30	1.383 (6)
Fe1—C4	2.361 (4)	C30—H30	0.9500
Fe1—C5	2.130 (3)	C30—C31	1.390 (6)
Fe1—C6	2.101 (4)	C31—H31	0.9500
Fe1—C7	2.065 (3)	C31—C32	1.387 (6)
Fe1—C8	2.137 (3)	C32—H32	0.9500
O1—C4	1.243 (5)	C33—C34	1.397 (6)
N1—C1	1.162 (6)	C33—C39	1.403 (5)
N1—C33	1.392 (5)	C34—C35	1.496 (6)
N2—C2	1.169 (5)	C34—C36	1.397 (6)
N2—C41	1.403 (6)	C35—H35A	0.9800
N3—C3	1.162 (4)	C35—H35B	0.9800
N3—C49	1.403 (4)	C35—H35C	0.9800
C4—C5	1.475 (6)	C36—H36	0.9500
C4—C8	1.487 (4)	C36—C37	1.386 (7)
C5—C6	1.438 (5)	C37—H37	0.9500
C5—C9	1.487 (4)	C37—C38	1.375 (7)
C6—C7	1.447 (5)	C38—H38	0.9500
C6—C15	1.482 (6)	C38—C39	1.384 (6)
C7—C8	1.434 (6)	C39—C40	1.501 (7)
C7—C21	1.494 (5)	C40—H40A	0.9800
C8—C27	1.478 (5)	C40—H40B	0.9800
C9—C10	1.388 (5)	C40—H40C	0.9800
C9—C14	1.402 (5)	C41—C42	1.397 (5)
C10—H10	0.9500	C41—C47	1.398 (6)
C10—C11	1.390 (5)	C42—C43	1.490 (6)
C11—H11	0.9500	C42—C44	1.392 (6)
C11—C12	1.387 (6)	C43—H43A	0.9800
C12—H12	0.9500	C43—H43B	0.9800
C12—C13	1.387 (6)	C43—H43C	0.9800
C13—H13	0.9500	C44—H44	0.9500
C13—C14	1.387 (5)	C44—C45	1.386 (8)
C14—H14	0.9500	C45—H45	0.9500
C15—C16	1.393 (5)	C45—C46	1.383 (8)
C15—C20	1.404 (5)	C46—H46	0.9500
C16—H16	0.9500	C46—C47	1.383 (7)
C16—C17	1.390 (6)	C47—C48	1.507 (7)
C17—H17	0.9500	C48—H48A	0.9800
C17—C18	1.383 (6)	C48—H48B	0.9800
C18—H18	0.9500	C48—H48C	0.9800
C18—C19	1.390 (6)	C49—C50	1.391 (5)
C19—H19	0.9500	C49—C55	1.404 (6)
C19—C20	1.389 (6)	C50—C51	1.498 (6)
C20—H20	0.9500	C50—C52	1.396 (5)
C21—C22	1.396 (5)	C51—H51A	0.9800
C21—C26	1.394 (5)	C51—H51B	0.9800
C22—H22	0.9500	C51—H51C	0.9800
C22—C23	1.390 (5)	C52—H52	0.9500
C23—H23	0.9500	C52—C53	1.387 (7)
C23—C24	1.396 (6)	C53—H53	0.9500
C24—H24	0.9500	C53—C54	1.377 (6)
C24—C25	1.386 (6)	C54—H54	0.9500
C25—H25	0.9500	C54—C55	1.393 (5)
C25—C26	1.388 (5)	C55—C56	1.493 (6)
C26—H26	0.9500	C56—H56A	0.9800
C27—C28	1.414 (5)	C56—H56B	0.9800
C27—C32	1.398 (6)	C56—H56C	0.9800
C28—H28	0.9500		
			
C1—Fe1—C4	81.84 (15)	C24—C25—C26	120.3 (4)
C1—Fe1—C5	98.56 (15)	C26—C25—H25	119.9
C1—Fe1—C6	138.28 (15)	C21—C26—H26	119.7
C1—Fe1—C7	140.51 (16)	C25—C26—C21	120.6 (4)
C1—Fe1—C8	100.74 (16)	C25—C26—H26	119.7
C2—Fe1—C1	98.02 (16)	C28—C27—C8	122.0 (3)
C2—Fe1—C4	137.55 (14)	C32—C27—C8	120.7 (3)
C2—Fe1—C5	101.46 (16)	C32—C27—C28	117.2 (3)
C2—Fe1—C6	92.42 (16)	C27—C28—H28	119.5
C2—Fe1—C7	120.43 (17)	C29—C28—C27	121.1 (4)
C2—Fe1—C8	158.98 (16)	C29—C28—H28	119.5
C3—Fe1—C1	92.11 (17)	C28—C29—H29	119.8
C3—Fe1—C2	92.78 (17)	C30—C29—C28	120.5 (4)
C3—Fe1—C4	129.67 (15)	C30—C29—H29	119.8
C3—Fe1—C5	160.77 (14)	C29—C30—H30	120.3
C3—Fe1—C6	127.73 (15)	C29—C30—C31	119.4 (4)
C3—Fe1—C7	94.48 (14)	C31—C30—H30	120.3
C3—Fe1—C8	95.90 (14)	C30—C31—H31	119.8
C5—Fe1—C4	37.91 (15)	C32—C31—C30	120.3 (4)
C5—Fe1—C8	66.48 (13)	C32—C31—H31	119.8
C6—Fe1—C4	63.99 (14)	C27—C32—H32	119.3
C6—Fe1—C5	39.73 (14)	C31—C32—C27	121.4 (3)
C6—Fe1—C8	67.18 (14)	C31—C32—H32	119.3
C7—Fe1—C4	64.22 (13)	N1—C33—C34	118.9 (4)
C7—Fe1—C5	67.20 (12)	N1—C33—C39	117.8 (4)
C7—Fe1—C6	40.63 (13)	C34—C33—C39	123.3 (4)
C7—Fe1—C8	39.85 (15)	C33—C34—C35	120.4 (4)
C8—Fe1—C4	38.22 (12)	C33—C34—C36	117.0 (4)
C1—N1—C33	178.6 (4)	C36—C34—C35	122.6 (4)
C2—N2—C41	159.5 (4)	C34—C35—H35A	109.5
C3—N3—C49	163.5 (4)	C34—C35—H35B	109.5
N1—C1—Fe1	177.1 (4)	C34—C35—H35C	109.5
N2—C2—Fe1	178.1 (4)	H35A—C35—H35B	109.5
N3—C3—Fe1	177.1 (4)	H35A—C35—H35C	109.5
O1—C4—Fe1	135.1 (3)	H35B—C35—H35C	109.5
O1—C4—C5	126.5 (3)	C34—C36—H36	119.7
O1—C4—C8	129.0 (3)	C37—C36—C34	120.5 (4)
C5—C4—Fe1	62.53 (18)	C37—C36—H36	119.7
C5—C4—C8	104.3 (3)	C36—C37—H37	119.6
C8—C4—Fe1	62.74 (18)	C38—C37—C36	120.8 (4)
C4—C5—Fe1	79.56 (19)	C38—C37—H37	119.6
C4—C5—C9	122.3 (3)	C37—C38—H38	119.4
C6—C5—Fe1	69.06 (18)	C37—C38—C39	121.2 (4)
C6—C5—C4	109.2 (3)	C39—C38—H38	119.4
C6—C5—C9	126.5 (4)	C33—C39—C40	120.5 (4)
C9—C5—Fe1	130.8 (2)	C38—C39—C33	117.0 (4)
C5—C6—Fe1	71.21 (19)	C38—C39—C40	122.4 (4)
C5—C6—C7	107.2 (3)	C39—C40—H40A	109.5
C5—C6—C15	127.0 (3)	C39—C40—H40B	109.5
C7—C6—Fe1	68.35 (19)	C39—C40—H40C	109.5
C7—C6—C15	125.4 (3)	H40A—C40—H40B	109.5
C15—C6—Fe1	131.2 (3)	H40A—C40—H40C	109.5
C6—C7—Fe1	71.02 (19)	H40B—C40—H40C	109.5
C6—C7—C21	122.3 (3)	C42—C41—N2	117.6 (4)
C8—C7—Fe1	72.8 (2)	C42—C41—C47	123.1 (4)
C8—C7—C6	109.0 (3)	C47—C41—N2	119.1 (4)
C8—C7—C21	128.1 (3)	C41—C42—C43	121.4 (4)
C21—C7—Fe1	129.3 (2)	C44—C42—C41	117.5 (4)
C4—C8—Fe1	79.0 (2)	C44—C42—C43	121.1 (4)
C7—C8—Fe1	67.4 (2)	C42—C43—H43A	109.5
C7—C8—C4	108.1 (3)	C42—C43—H43B	109.5
C7—C8—C27	125.8 (3)	C42—C43—H43C	109.5
C27—C8—Fe1	129.2 (3)	H43A—C43—H43B	109.5
C27—C8—C4	124.8 (3)	H43A—C43—H43C	109.5
C10—C9—C5	121.4 (3)	H43B—C43—H43C	109.5
C10—C9—C14	118.0 (3)	C42—C44—H44	119.8
C14—C9—C5	120.6 (3)	C45—C44—C42	120.4 (4)
C9—C10—H10	119.4	C45—C44—H44	119.8
C9—C10—C11	121.1 (4)	C44—C45—H45	119.7
C11—C10—H10	119.4	C46—C45—C44	120.6 (5)
C10—C11—H11	119.9	C46—C45—H45	119.7
C12—C11—C10	120.3 (4)	C45—C46—H46	119.4
C12—C11—H11	119.9	C45—C46—C47	121.1 (5)
C11—C12—H12	120.3	C47—C46—H46	119.4
C11—C12—C13	119.4 (3)	C41—C47—C48	119.8 (4)
C13—C12—H12	120.3	C46—C47—C41	117.3 (4)
C12—C13—H13	119.9	C46—C47—C48	122.9 (5)
C14—C13—C12	120.2 (4)	C47—C48—H48A	109.5
C14—C13—H13	119.9	C47—C48—H48B	109.5
C9—C14—H14	119.5	C47—C48—H48C	109.5
C13—C14—C9	121.1 (4)	H48A—C48—H48B	109.5
C13—C14—H14	119.5	H48A—C48—H48C	109.5
C16—C15—C6	123.4 (3)	H48B—C48—H48C	109.5
C16—C15—C20	118.0 (4)	N3—C49—C55	117.9 (3)
C20—C15—C6	118.4 (3)	C50—C49—N3	118.5 (4)
C15—C16—H16	119.4	C50—C49—C55	123.5 (3)
C17—C16—C15	121.1 (4)	C49—C50—C51	120.1 (3)
C17—C16—H16	119.4	C49—C50—C52	117.2 (4)
C16—C17—H17	119.8	C52—C50—C51	122.7 (4)
C18—C17—C16	120.4 (4)	C50—C51—H51A	109.5
C18—C17—H17	119.8	C50—C51—H51B	109.5
C17—C18—H18	120.4	C50—C51—H51C	109.5
C17—C18—C19	119.2 (4)	H51A—C51—H51B	109.5
C19—C18—H18	120.4	H51A—C51—H51C	109.5
C18—C19—H19	119.7	H51B—C51—H51C	109.5
C20—C19—C18	120.6 (4)	C50—C52—H52	119.6
C20—C19—H19	119.7	C53—C52—C50	120.7 (4)
C15—C20—H20	119.7	C53—C52—H52	119.6
C19—C20—C15	120.6 (4)	C52—C53—H53	119.7
C19—C20—H20	119.7	C54—C53—C52	120.5 (4)
C22—C21—C7	121.6 (3)	C54—C53—H53	119.7
C26—C21—C7	119.3 (3)	C53—C54—H54	119.4
C26—C21—C22	118.8 (3)	C53—C54—C55	121.3 (4)
C21—C22—H22	119.6	C55—C54—H54	119.4
C23—C22—C21	120.7 (4)	C49—C55—C56	120.8 (4)
C23—C22—H22	119.6	C54—C55—C49	116.7 (4)
C22—C23—H23	120.1	C54—C55—C56	122.4 (4)
C22—C23—C24	119.8 (4)	C55—C56—H56A	109.5
C24—C23—H23	120.1	C55—C56—H56B	109.5
C23—C24—H24	120.2	C55—C56—H56C	109.5
C25—C24—C23	119.7 (4)	H56A—C56—H56B	109.5
C25—C24—H24	120.2	H56A—C56—H56C	109.5
C24—C25—H25	119.9	H56B—C56—H56C	109.5
			
Fe1—C4—C5—C6	63.3 (2)	C7—C21—C26—C25	173.5 (3)
Fe1—C4—C5—C9	−132.0 (3)	C8—C4—C5—Fe1	−48.3 (2)
Fe1—C4—C8—C7	−61.6 (2)	C8—C4—C5—C6	15.0 (3)
Fe1—C4—C8—C27	130.5 (4)	C8—C4—C5—C9	179.7 (3)
Fe1—C5—C6—C7	59.1 (2)	C8—C7—C21—C22	−132.2 (4)
Fe1—C5—C6—C15	−128.0 (4)	C8—C7—C21—C26	53.1 (5)
Fe1—C5—C9—C10	−51.3 (6)	C8—C27—C28—C29	−173.0 (4)
Fe1—C5—C9—C14	131.7 (4)	C8—C27—C32—C31	174.1 (4)
Fe1—C6—C7—C8	63.3 (2)	C9—C5—C6—Fe1	125.9 (3)
Fe1—C6—C7—C21	−125.1 (3)	C9—C5—C6—C7	−175.0 (3)
Fe1—C6—C15—C16	−49.1 (5)	C9—C5—C6—C15	−2.1 (6)
Fe1—C6—C15—C20	135.7 (3)	C9—C10—C11—C12	−0.4 (6)
Fe1—C7—C8—C4	69.3 (2)	C10—C9—C14—C13	0.9 (6)
Fe1—C7—C8—C27	−122.9 (4)	C10—C11—C12—C13	1.2 (6)
Fe1—C7—C21—C22	−33.2 (5)	C11—C12—C13—C14	−1.0 (6)
Fe1—C7—C21—C26	152.1 (3)	C12—C13—C14—C9	−0.1 (6)
Fe1—C8—C27—C28	−61.0 (5)	C14—C9—C10—C11	−0.7 (6)
Fe1—C8—C27—C32	122.9 (4)	C15—C6—C7—Fe1	126.0 (3)
O1—C4—C5—Fe1	127.5 (4)	C15—C6—C7—C8	−170.7 (3)
O1—C4—C5—C6	−169.2 (4)	C15—C6—C7—C21	0.9 (5)
O1—C4—C5—C9	−4.5 (5)	C15—C16—C17—C18	2.3 (5)
O1—C4—C8—Fe1	−127.4 (4)	C16—C15—C20—C19	0.2 (5)
O1—C4—C8—C7	171.0 (4)	C16—C17—C18—C19	0.0 (5)
O1—C4—C8—C27	3.0 (6)	C17—C18—C19—C20	−2.2 (6)
N1—C33—C34—C35	−1.1 (5)	C18—C19—C20—C15	2.1 (5)
N1—C33—C34—C36	179.6 (3)	C20—C15—C16—C17	−2.3 (5)
N1—C33—C39—C38	−178.8 (3)	C21—C7—C8—Fe1	126.9 (4)
N1—C33—C39—C40	0.6 (5)	C21—C7—C8—C4	−163.8 (3)
N2—C41—C42—C43	4.5 (6)	C21—C7—C8—C27	3.9 (6)
N2—C41—C42—C44	−173.3 (4)	C21—C22—C23—C24	−0.3 (6)
N2—C41—C47—C46	172.8 (4)	C22—C21—C26—C25	−1.3 (5)
N2—C41—C47—C48	−4.5 (6)	C22—C23—C24—C25	−1.6 (6)
N3—C49—C50—C51	−1.5 (5)	C23—C24—C25—C26	2.1 (6)
N3—C49—C50—C52	177.1 (3)	C24—C25—C26—C21	−0.6 (6)
N3—C49—C55—C54	−178.9 (4)	C26—C21—C22—C23	1.8 (6)
N3—C49—C55—C56	−2.1 (6)	C27—C28—C29—C30	−2.1 (6)
C2—N2—C41—C42	101.2 (11)	C28—C27—C32—C31	−2.1 (6)
C2—N2—C41—C47	−74.3 (12)	C28—C29—C30—C31	−0.4 (7)
C3—N3—C49—C50	−175.2 (11)	C29—C30—C31—C32	1.5 (7)
C3—N3—C49—C55	2.9 (14)	C30—C31—C32—C27	−0.2 (7)
C4—C5—C6—Fe1	−70.2 (2)	C32—C27—C28—C29	3.3 (6)
C4—C5—C6—C7	−11.1 (4)	C33—C34—C36—C37	−1.3 (5)
C4—C5—C6—C15	161.8 (3)	C34—C33—C39—C38	0.5 (5)
C4—C5—C9—C10	53.9 (5)	C34—C33—C39—C40	179.9 (4)
C4—C5—C9—C14	−123.1 (4)	C34—C36—C37—C38	1.4 (6)
C4—C8—C27—C28	−166.6 (3)	C35—C34—C36—C37	179.4 (4)
C4—C8—C27—C32	17.3 (6)	C36—C37—C38—C39	−0.5 (6)
C5—C4—C8—Fe1	48.2 (2)	C37—C38—C39—C33	−0.4 (5)
C5—C4—C8—C7	−13.4 (4)	C37—C38—C39—C40	−179.8 (4)
C5—C4—C8—C27	178.7 (3)	C39—C33—C34—C35	179.7 (4)
C5—C6—C7—Fe1	−60.9 (2)	C39—C33—C34—C36	0.3 (5)
C5—C6—C7—C8	2.4 (4)	C41—C42—C44—C45	−0.2 (6)
C5—C6—C7—C21	173.9 (3)	C42—C41—C47—C46	−2.5 (6)
C5—C6—C15—C16	48.0 (5)	C42—C41—C47—C48	−179.8 (4)
C5—C6—C15—C20	−127.2 (4)	C42—C44—C45—C46	−1.1 (7)
C5—C9—C10—C11	−177.7 (4)	C43—C42—C44—C45	−178.0 (4)
C5—C9—C14—C13	178.0 (4)	C44—C45—C46—C47	0.7 (7)
C6—C5—C9—C10	−144.1 (4)	C45—C46—C47—C41	1.1 (7)
C6—C5—C9—C14	38.8 (5)	C45—C46—C47—C48	178.3 (5)
C6—C7—C8—Fe1	−62.2 (2)	C47—C41—C42—C43	179.8 (4)
C6—C7—C8—C4	7.1 (4)	C47—C41—C42—C44	2.1 (6)
C6—C7—C8—C27	174.9 (3)	C49—C50—C52—C53	2.3 (5)
C6—C7—C21—C22	58.0 (5)	C50—C49—C55—C54	−0.9 (6)
C6—C7—C21—C26	−116.8 (4)	C50—C49—C55—C56	176.0 (4)
C6—C15—C16—C17	−177.6 (3)	C50—C52—C53—C54	−1.8 (6)
C6—C15—C20—C19	175.7 (3)	C51—C50—C52—C53	−179.2 (4)
C7—C6—C15—C16	−140.3 (4)	C52—C53—C54—C55	−0.2 (6)
C7—C6—C15—C20	44.5 (5)	C53—C54—C55—C49	1.4 (6)
C7—C8—C27—C28	27.6 (6)	C53—C54—C55—C56	−175.3 (4)
C7—C8—C27—C32	−148.5 (4)	C55—C49—C50—C51	−179.6 (4)
C7—C21—C22—C23	−173.0 (3)	C55—C49—C50—C52	−1.0 (5)

### Tris(naphthalen-2-yl isocyanide)(η4-tetraphenylcyclopentadienone)iron acetone hemisolvate (twin) Crystal data

**Table:**

[Fe(C_11_H_7_N)_3_(C_29_H_20_O)]_2_·C_3_H_6_O	*F*(000) = 1936
*M**_r_* = 1857.73	*D*_x_ = 1.326 Mg m^−^^3^
Monoclinic, *P*2_1_	Cu *K*α radiation, λ = 1.54184 Å
*a* = 15.2471 (1) Å	Cell parameters from 61660 reflections
*b* = 19.8604 (1) Å	θ = 2.9–78.4°
*c* = 15.5785 (1) Å	µ = 2.98 mm^−^^1^
β = 99.378 (1)°	*T* = 100 K
*V* = 4654.33 (5) Å^3^	Needle, clear colourless
*Z* = 2	0.18 × 0.07 × 0.05 mm

### Tris(naphthalen-2-yl isocyanide)(η4-tetraphenylcyclopentadienone)iron acetone hemisolvate (twin) Data collection

**Table:**

XtaLAB Synergy, Dualflex, Pilatus 300K diffractometer	18788 independent reflections
Radiation source: micro-focus sealed X-ray tube, PhotonJet (Cu) X-ray Source	17761 reflections with *I* > 2σ(*I*)
Mirror monochromator	*R*_int_ = 0.050
Detector resolution: 5.8140 pixels mm^-1^	θ_max_ = 78.8°, θ_min_ = 2.9°
ω scans	*h* = −19→19
Absorption correction: gaussian (CrysAlisPro; Rigaku OD, 2021)	*k* = −23→24
*T*_min_ = 0.779, *T*_max_ = 1.000	*l* = −19→19
89473 measured reflections	

### Tris(naphthalen-2-yl isocyanide)(η4-tetraphenylcyclopentadienone)iron acetone hemisolvate (twin) Refinement

**Table:**

Refinement on *F*^2^	Hydrogen site location: inferred from neighbouring sites
Least-squares matrix: full	H-atom parameters constrained
*R*[*F*^2^ > 2σ(*F*^2^)] = 0.036	*w* = 1/[σ^2^(*F*_o_^2^) + (0.0732*P*)^2^ + 0.229*P*] where *P* = (*F*_o_^2^ + 2*F*_c_^2^)/3
*wR*(*F*^2^) = 0.100	(Δ/σ)_max_ = 0.001
*S* = 1.07	Δρ_max_ = 0.28 e Å^−^^3^
18788 reflections	Δρ_min_ = −0.37 e Å^−^^3^
1390 parameters	Absolute structure: Classical Flack (1983) method preferred over Parsons because s.u. lower
1606 restraints	Absolute structure parameter: −0.004 (2)
Primary atom site location: dual	

### Tris(naphthalen-2-yl isocyanide)(η4-tetraphenylcyclopentadienone)iron acetone hemisolvate (twin) Special details

**Table:**

Geometry. All esds (except the esd in the dihedral angle between two l.s. planes) are estimated using the full covariance matrix. The cell esds are taken into account individually in the estimation of esds in distances, angles and torsion angles; correlations between esds in cell parameters are only used when they are defined by crystal symmetry. An approximate (isotropic) treatment of cell esds is used for estimating esds involving l.s. planes.
Refinement. Twinned specimen. De-twinned HKLF4 gave best results.

### Tris(naphthalen-2-yl isocyanide)(η4-tetraphenylcyclopentadienone)iron acetone hemisolvate (twin) Fractional atomic coordinates and isotropic or equivalent isotropic displacement parameters (Å^2^)

**Table:**

	*x*	*y*	*z*	*U*_iso_*/*U*_eq_	Occ. (<1)
Fe1A	0.57957 (3)	0.45750 (2)	0.58041 (3)	0.01517 (10)	
O1A	0.51344 (13)	0.30215 (10)	0.51440 (13)	0.0201 (4)	
N1A	0.67302 (16)	0.34102 (13)	0.67723 (16)	0.0224 (5)	
N2A	0.54485 (17)	0.53463 (13)	0.73647 (17)	0.0243 (5)	
N3A	0.73794 (16)	0.53525 (14)	0.54475 (17)	0.0235 (5)	
C1A	0.63882 (18)	0.38791 (15)	0.64210 (18)	0.0182 (5)	
C2A	0.56081 (18)	0.50575 (14)	0.67578 (18)	0.0188 (5)	
C3A	0.67717 (19)	0.50556 (14)	0.56222 (18)	0.0191 (5)	
C4A	0.50553 (18)	0.36441 (15)	0.50618 (18)	0.0194 (6)	
C5A	0.55382 (17)	0.41021 (14)	0.45526 (17)	0.0172 (5)	
C6A	0.51530 (17)	0.47630 (14)	0.45515 (17)	0.0170 (5)	
C7A	0.45269 (17)	0.47642 (14)	0.51526 (17)	0.0172 (5)	
C8A	0.45225 (17)	0.40968 (14)	0.55248 (17)	0.0172 (5)	
C9A	0.70183 (19)	0.27731 (15)	0.70810 (18)	0.0199 (5)	
C10A	0.6534 (2)	0.22088 (17)	0.6712 (2)	0.0241 (6)	
H10A	0.604652	0.226301	0.625213	0.029*	
C11A	0.6779 (2)	0.15860 (16)	0.7027 (2)	0.0248 (6)	
H11A	0.646115	0.120231	0.677981	0.030*	
C12A	0.75027 (19)	0.14989 (15)	0.77214 (19)	0.0217 (6)	
C13A	0.7735 (2)	0.08573 (17)	0.8091 (2)	0.0304 (7)	
H13A	0.740520	0.047119	0.786928	0.036*	
C14A	0.8428 (2)	0.07900 (18)	0.8762 (2)	0.0339 (7)	
H14A	0.856863	0.035855	0.900958	0.041*	
C15A	0.8936 (2)	0.13552 (18)	0.9090 (2)	0.0282 (7)	
H15A	0.942079	0.130263	0.955290	0.034*	
C16A	0.87303 (19)	0.19785 (16)	0.8741 (2)	0.0234 (6)	
H16A	0.908102	0.235582	0.895785	0.028*	
C17A	0.79993 (18)	0.20695 (15)	0.80594 (19)	0.0190 (5)	
C18A	0.77404 (18)	0.27145 (15)	0.77292 (18)	0.0195 (5)	
H18A	0.806232	0.310309	0.795322	0.023*	
C19A	0.51160 (19)	0.56466 (15)	0.80555 (19)	0.0209 (6)	
C20A	0.56227 (19)	0.56089 (16)	0.8899 (2)	0.0231 (6)	
H20A	0.619182	0.540154	0.898781	0.028*	
C21A	0.5283 (2)	0.58748 (16)	0.9583 (2)	0.0247 (6)	
H21A	0.562294	0.585714	1.015100	0.030*	
C22A	0.4429 (2)	0.61767 (15)	0.9458 (2)	0.0236 (6)	
C23A	0.4047 (2)	0.64311 (18)	1.0168 (2)	0.0328 (7)	
H23A	0.437245	0.640741	1.074214	0.039*	
C24A	0.3217 (3)	0.6709 (2)	1.0030 (3)	0.0431 (9)	
H24A	0.296726	0.687512	1.050820	0.052*	
C25A	0.2729 (3)	0.6750 (2)	0.9187 (3)	0.0473 (10)	
H25A	0.215342	0.694618	0.910039	0.057*	
C26A	0.3074 (2)	0.6511 (2)	0.8482 (3)	0.0369 (8)	
H26A	0.273691	0.654116	0.791436	0.044*	
C27A	0.3935 (2)	0.62181 (16)	0.8609 (2)	0.0243 (6)	
C28A	0.43005 (19)	0.59519 (16)	0.7902 (2)	0.0237 (6)	
H28A	0.398284	0.598495	0.732556	0.028*	
C29A	0.80196 (19)	0.56989 (16)	0.50659 (19)	0.0217 (6)	
C30A	0.8045 (2)	0.64098 (17)	0.5122 (2)	0.0258 (6)	
H30A	0.765661	0.664522	0.543548	0.031*	
C31A	0.8643 (2)	0.67528 (17)	0.4715 (2)	0.0291 (7)	
H31A	0.866065	0.723057	0.474331	0.035*	
C32A	0.9232 (2)	0.64074 (18)	0.4254 (2)	0.0272 (7)	
C33A	0.9860 (2)	0.6753 (2)	0.3829 (2)	0.0396 (9)	
H33A	0.990077	0.722974	0.386526	0.048*	
C34A	1.0404 (2)	0.6406 (3)	0.3372 (3)	0.0486 (11)	
H34A	1.081593	0.664290	0.308811	0.058*	
C35A	1.0356 (2)	0.5696 (3)	0.3317 (3)	0.0463 (10)	
H35A	1.073381	0.545934	0.299303	0.056*	
C36A	0.9775 (2)	0.5352 (2)	0.3725 (2)	0.0351 (8)	
H36A	0.975569	0.487424	0.368836	0.042*	
C37A	0.91958 (19)	0.56911 (18)	0.42043 (19)	0.0246 (6)	
C38A	0.85803 (19)	0.53476 (16)	0.4627 (2)	0.0233 (6)	
H38A	0.855382	0.486981	0.460808	0.028*	
C39A	0.61525 (18)	0.38679 (15)	0.39642 (18)	0.0193 (5)	
C40A	0.67738 (18)	0.42953 (15)	0.36753 (19)	0.0207 (6)	
H40A	0.683802	0.474357	0.388900	0.025*	
C41A	0.7300 (2)	0.40735 (17)	0.3079 (2)	0.0259 (6)	
H41A	0.771436	0.437220	0.288756	0.031*	
C42A	0.7221 (2)	0.34175 (18)	0.2764 (2)	0.0285 (7)	
H42A	0.757937	0.326560	0.235712	0.034*	
C43A	0.6613 (2)	0.29889 (17)	0.3051 (2)	0.0284 (7)	
H43A	0.655601	0.253943	0.284034	0.034*	
C44A	0.6086 (2)	0.32084 (16)	0.3642 (2)	0.0235 (6)	
H44A	0.567307	0.290659	0.383178	0.028*	
C45A	0.52444 (18)	0.53034 (15)	0.39123 (19)	0.0198 (5)	
C46A	0.55581 (19)	0.59498 (16)	0.4140 (2)	0.0230 (6)	
H46A	0.574176	0.605952	0.473555	0.028*	
C47A	0.5606 (2)	0.64365 (17)	0.3505 (2)	0.0287 (7)	
H47A	0.581106	0.687717	0.366764	0.034*	
C48A	0.5352 (2)	0.62750 (18)	0.2634 (2)	0.0307 (7)	
H48A	0.538339	0.660522	0.219918	0.037*	
C49A	0.5052 (2)	0.56322 (18)	0.2398 (2)	0.0279 (6)	
H49A	0.488549	0.552140	0.180053	0.033*	
C50A	0.49930 (19)	0.51486 (16)	0.30310 (19)	0.0224 (6)	
H50A	0.478080	0.471039	0.286393	0.027*	
C51A	0.38688 (18)	0.53063 (15)	0.52111 (18)	0.0182 (5)	
C52A	0.29681 (19)	0.51478 (16)	0.49939 (19)	0.0217 (6)	
H52A	0.279400	0.469455	0.486776	0.026*	
C53A	0.2320 (2)	0.56482 (18)	0.4960 (2)	0.0272 (6)	
H53A	0.170942	0.553425	0.480824	0.033*	
C54A	0.2565 (2)	0.63095 (18)	0.5147 (2)	0.0303 (7)	
H54A	0.212541	0.665145	0.511339	0.036*	
C55A	0.3452 (2)	0.64695 (16)	0.5382 (2)	0.0280 (7)	
H55A	0.362133	0.692280	0.551516	0.034*	
C56A	0.4103 (2)	0.59708 (15)	0.54263 (19)	0.0224 (6)	
H56A	0.471029	0.608517	0.560455	0.027*	
C57A	0.39119 (18)	0.38613 (15)	0.61099 (18)	0.0190 (5)	
C58A	0.35409 (19)	0.32133 (15)	0.5997 (2)	0.0221 (6)	
H58A	0.370876	0.292262	0.556750	0.026*	
C59A	0.2930 (2)	0.29936 (17)	0.6509 (2)	0.0277 (7)	
H59A	0.268651	0.255334	0.642781	0.033*	
C60A	0.2672 (2)	0.34098 (18)	0.7136 (2)	0.0287 (7)	
H60A	0.225122	0.325909	0.748065	0.034*	
C61A	0.3038 (2)	0.40517 (18)	0.7255 (2)	0.0265 (6)	
H61A	0.286521	0.434007	0.768500	0.032*	
C62A	0.36511 (19)	0.42737 (16)	0.67538 (19)	0.0216 (6)	
H62A	0.389856	0.471177	0.684733	0.026*	
Fe1B	0.83999 (3)	0.48533 (2)	0.86648 (3)	0.01583 (10)	
O1B	0.92081 (13)	0.63902 (11)	0.92958 (13)	0.0213 (4)	
N1B	0.74010 (16)	0.60604 (13)	0.78767 (17)	0.0225 (5)	
N2B	0.68892 (17)	0.39653 (13)	0.89964 (18)	0.0250 (5)	
N3B	0.85773 (17)	0.43202 (14)	0.69046 (16)	0.0239 (5)	
C1B	0.77407 (17)	0.55748 (15)	0.81883 (18)	0.0188 (5)	
C2B	0.74839 (19)	0.43019 (15)	0.88633 (18)	0.0196 (5)	
C3B	0.84823 (17)	0.45049 (15)	0.75983 (18)	0.0198 (5)	
C4B	0.92462 (18)	0.57659 (15)	0.93463 (18)	0.0194 (6)	
C5B	0.96763 (17)	0.52974 (15)	0.88028 (18)	0.0184 (5)	
C6B	0.97052 (17)	0.46366 (15)	0.91895 (17)	0.0186 (5)	
C7B	0.91703 (17)	0.46555 (15)	0.98714 (17)	0.0178 (5)	
C8B	0.88065 (18)	0.53202 (15)	0.99053 (18)	0.0188 (5)	
C9B	0.72516 (19)	0.67210 (15)	0.75815 (19)	0.0209 (6)	
C10B	0.65394 (19)	0.68525 (16)	0.6900 (2)	0.0240 (6)	
H10B	0.616139	0.649723	0.665635	0.029*	
C11B	0.6399 (2)	0.74952 (17)	0.6594 (2)	0.0243 (6)	
H11B	0.592032	0.758444	0.613650	0.029*	
C12B	0.6961 (2)	0.80323 (16)	0.6950 (2)	0.0235 (6)	
C13B	0.6836 (2)	0.87017 (17)	0.6651 (2)	0.0282 (7)	
H13B	0.634660	0.880663	0.621153	0.034*	
C14B	0.7408 (2)	0.92001 (17)	0.6984 (2)	0.0314 (7)	
H14B	0.731748	0.964686	0.676944	0.038*	
C15B	0.8133 (2)	0.90552 (18)	0.7645 (2)	0.0306 (7)	
H15B	0.853157	0.940443	0.786874	0.037*	
C16B	0.8265 (2)	0.84174 (17)	0.7966 (2)	0.0266 (6)	
H16B	0.875198	0.832655	0.841453	0.032*	
C17B	0.76811 (19)	0.78889 (15)	0.76337 (19)	0.0213 (6)	
C18B	0.78143 (19)	0.72194 (16)	0.7949 (2)	0.0218 (6)	
H18B	0.828619	0.711806	0.840886	0.026*	
C19B	0.6131 (2)	0.35925 (18)	0.9116 (3)	0.0221 (7)	0.911 (3)
C20B	0.5515 (2)	0.33995 (18)	0.8379 (2)	0.0248 (7)	0.911 (3)
H20B	0.560941	0.352261	0.781151	0.030*	0.911 (3)
C21B	0.4783 (2)	0.30340 (18)	0.8490 (2)	0.0271 (7)	0.911 (3)
H21B	0.437479	0.289478	0.799411	0.032*	0.911 (3)
C22B	0.4622 (2)	0.28597 (17)	0.9331 (2)	0.0254 (7)	0.911 (3)
C23B	0.3855 (2)	0.2489 (2)	0.9462 (3)	0.0303 (8)	0.911 (3)
H23B	0.344887	0.233554	0.897237	0.036*	0.911 (3)
C24B	0.3695 (3)	0.2350 (2)	1.0284 (3)	0.0342 (9)	0.911 (3)
H24B	0.317937	0.210406	1.036257	0.041*	0.911 (3)
C25B	0.4295 (3)	0.2573 (2)	1.1013 (3)	0.0362 (9)	0.911 (3)
H25B	0.417843	0.248078	1.158200	0.043*	0.911 (3)
C26B	0.5045 (3)	0.2923 (2)	1.0910 (2)	0.0332 (8)	0.911 (3)
H26B	0.544671	0.306553	1.140757	0.040*	0.911 (3)
C27B	0.5228 (3)	0.3072 (2)	1.0069 (3)	0.0248 (8)	0.911 (3)
C28B	0.5998 (2)	0.34384 (18)	0.9947 (2)	0.0243 (7)	0.911 (3)
H28B	0.641734	0.357599	1.043476	0.029*	0.911 (3)
C29B	0.8895 (2)	0.40251 (19)	0.6196 (2)	0.0216 (8)	0.911 (3)
C30B	0.8312 (2)	0.36323 (18)	0.5594 (2)	0.0230 (7)	0.911 (3)
H30B	0.770749	0.357988	0.565929	0.028*	0.911 (3)
C31B	0.8630 (2)	0.33285 (18)	0.4917 (2)	0.0239 (7)	0.911 (3)
H31B	0.824236	0.306230	0.451373	0.029*	0.911 (3)
C32B	0.9529 (2)	0.34066 (18)	0.4811 (2)	0.0223 (7)	0.911 (3)
C33B	0.9873 (2)	0.30916 (19)	0.4119 (2)	0.0277 (7)	0.911 (3)
H33B	0.949890	0.281407	0.371841	0.033*	0.911 (3)
C34B	1.0743 (3)	0.3186 (2)	0.4026 (2)	0.0329 (8)	0.911 (3)
H34B	1.096570	0.297663	0.355705	0.039*	0.911 (3)
C35B	1.1307 (2)	0.3589 (2)	0.4614 (2)	0.0338 (8)	0.911 (3)
H35B	1.190824	0.364882	0.454062	0.041*	0.911 (3)
C36B	1.1002 (2)	0.3898 (2)	0.5292 (2)	0.0309 (8)	0.911 (3)
H36B	1.139090	0.416943	0.568682	0.037*	0.911 (3)
C37B	1.0105 (2)	0.3813 (2)	0.5407 (2)	0.0224 (7)	0.911 (3)
C38B	0.9766 (2)	0.4121 (2)	0.6107 (2)	0.0244 (7)	0.911 (3)
H38B	1.014153	0.439353	0.651268	0.029*	0.911 (3)
C39B	1.01292 (18)	0.55284 (15)	0.80852 (19)	0.0207 (6)	
C40B	1.0962 (2)	0.52835 (18)	0.7958 (2)	0.0321 (7)	
H40B	1.126573	0.496574	0.835617	0.039*	
C41B	1.1349 (2)	0.5500 (2)	0.7259 (3)	0.0418 (9)	
H41B	1.190619	0.532068	0.717528	0.050*	
C42B	1.0928 (2)	0.5974 (2)	0.6683 (3)	0.0396 (9)	
H42B	1.119921	0.612392	0.620926	0.048*	
C43B	1.0111 (2)	0.6230 (2)	0.6800 (2)	0.0363 (8)	
H43B	0.981738	0.655371	0.640591	0.044*	
C44B	0.9721 (2)	0.60103 (18)	0.7498 (2)	0.0286 (7)	
H44B	0.916392	0.619253	0.757747	0.034*	
C45B	1.03063 (18)	0.40615 (15)	0.90813 (18)	0.0188 (5)	
C46B	1.01554 (19)	0.35929 (16)	0.8411 (2)	0.0235 (6)	
H46B	0.966305	0.364944	0.795786	0.028*	
C47B	1.0717 (2)	0.30408 (17)	0.8396 (2)	0.0266 (6)	
H47B	1.060388	0.272563	0.793226	0.032*	
C48B	1.1436 (2)	0.29478 (16)	0.9048 (2)	0.0259 (6)	
H48B	1.181247	0.256750	0.903943	0.031*	
C49B	1.1602 (2)	0.34130 (18)	0.9711 (2)	0.0306 (7)	
H49B	1.210247	0.335789	1.015613	0.037*	
C50B	1.1042 (2)	0.39609 (17)	0.9733 (2)	0.0278 (7)	
H50B	1.116039	0.427377	1.019838	0.033*	
C51B	0.91505 (18)	0.41028 (15)	1.05049 (18)	0.0191 (5)	
C52B	0.9313 (2)	0.42455 (16)	1.13966 (19)	0.0236 (6)	
H52B	0.936576	0.469965	1.158958	0.028*	
C53B	0.9397 (2)	0.37234 (17)	1.2002 (2)	0.0274 (7)	
H53B	0.948924	0.382393	1.260626	0.033*	
C54B	0.9346 (2)	0.30613 (17)	1.1730 (2)	0.0272 (6)	
H54B	0.942342	0.270743	1.214536	0.033*	
C55B	0.9182 (2)	0.29127 (16)	1.0844 (2)	0.0253 (6)	
H55B	0.914824	0.245770	1.065358	0.030*	
C56B	0.9069 (2)	0.34332 (16)	1.02437 (19)	0.0231 (6)	
H56B	0.893207	0.333024	0.964118	0.028*	
C57B	0.82839 (19)	0.55533 (15)	1.05734 (18)	0.0203 (6)	
C58B	0.8589 (2)	0.60964 (16)	1.1105 (2)	0.0254 (6)	
H58B	0.908401	0.635041	1.098741	0.031*	
C59B	0.8177 (2)	0.62699 (17)	1.1804 (2)	0.0292 (7)	
H59B	0.840065	0.663567	1.216815	0.035*	
C60B	0.7441 (2)	0.59151 (17)	1.1979 (2)	0.0276 (6)	
H60B	0.716516	0.603317	1.246236	0.033*	
C61B	0.7112 (2)	0.53847 (18)	1.1437 (2)	0.0271 (6)	
H61B	0.660111	0.514472	1.154100	0.033*	
C62B	0.75330 (19)	0.52061 (16)	1.0743 (2)	0.0229 (6)	
H62B	0.730631	0.484204	1.037704	0.028*	
O1	0.49104 (17)	0.46261 (14)	0.07107 (18)	0.0412 (6)	
C1	0.4135 (2)	0.45163 (19)	0.0431 (2)	0.0345 (7)	
C2	0.3474 (3)	0.4399 (3)	0.1032 (3)	0.0576 (13)	
H2A	0.338023	0.391409	0.109046	0.086*	
H2B	0.290947	0.461557	0.079428	0.086*	
H2C	0.370223	0.459071	0.160499	0.086*	
C3	0.3779 (3)	0.4484 (2)	−0.0521 (2)	0.0429 (9)	
H3A	0.339416	0.487317	−0.068767	0.064*	
H3B	0.343598	0.406839	−0.064905	0.064*	
H3C	0.427457	0.448860	−0.085079	0.064*	
C29C	0.9079 (18)	0.4224 (17)	0.6256 (19)	0.026 (6)	0.089 (3)
C38C	0.866 (2)	0.3738 (16)	0.5692 (19)	0.0230 (7)	0.089 (3)
H38C	0.806871	0.359483	0.571348	0.028*	0.089 (3)
C37C	0.9160 (17)	0.3467 (11)	0.5082 (14)	0.020 (5)	0.089 (3)
C36C	0.8793 (19)	0.2968 (13)	0.4492 (17)	0.021 (5)	0.089 (3)
H36C	0.820539	0.281090	0.449957	0.025*	0.089 (3)
C35C	0.9295 (18)	0.2701 (15)	0.3890 (18)	0.0277 (7)	0.089 (3)
H35C	0.904696	0.237224	0.347822	0.033*	0.089 (3)
C34C	1.0173 (19)	0.2930 (17)	0.391 (2)	0.0277 (7)	0.089 (3)
H34C	1.051866	0.271978	0.353096	0.033*	0.089 (3)
C33C	1.0567 (19)	0.3446 (16)	0.4447 (17)	0.026 (5)	0.089 (3)
H33C	1.113704	0.362489	0.440700	0.031*	0.089 (3)
C32C	1.0038 (18)	0.3678 (16)	0.5059 (19)	0.021 (6)	0.089 (3)
C31C	1.042 (2)	0.4187 (15)	0.5635 (18)	0.023 (5)	0.089 (3)
H31C	1.099956	0.435503	0.559880	0.028*	0.089 (3)
C30C	0.9947 (17)	0.4440 (17)	0.6259 (18)	0.022 (6)	0.089 (3)
H30C	1.021361	0.475551	0.668029	0.027*	0.089 (3)
C19C	0.6385 (18)	0.3566 (15)	0.9468 (16)	0.022 (5)	0.089 (3)
C27C	0.5010 (14)	0.3005 (11)	0.9272 (17)	0.035 (6)	0.089 (3)
C25C	0.3569 (18)	0.2418 (15)	0.906 (2)	0.037 (6)	0.089 (3)
H25C	0.306747	0.224472	0.867854	0.044*	0.089 (3)
C20C	0.6507 (16)	0.3508 (15)	1.0366 (16)	0.0243 (7)	0.089 (3)
H20C	0.701562	0.368029	1.073340	0.029*	0.089 (3)
C24C	0.366 (2)	0.2352 (17)	0.995 (2)	0.0342 (9)	0.089 (3)
H24C	0.320658	0.212152	1.018728	0.041*	0.089 (3)
C21C	0.5822 (15)	0.3176 (15)	1.0686 (18)	0.027 (5)	0.089 (3)
H21C	0.587951	0.312112	1.129876	0.032*	0.089 (3)
C26C	0.4252 (16)	0.2752 (14)	0.8738 (17)	0.0254 (7)	0.089 (3)
H26C	0.420284	0.281098	0.812709	0.030*	0.089 (3)
C22C	0.5054 (17)	0.2917 (18)	1.0172 (18)	0.028 (7)	0.089 (3)
C28C	0.5660 (19)	0.3333 (16)	0.8884 (18)	0.025 (6)	0.089 (3)
H28C	0.561018	0.339066	0.827228	0.030*	0.089 (3)
C23C	0.4378 (17)	0.2604 (17)	1.0536 (19)	0.034 (6)	0.089 (3)
H23C	0.440667	0.256530	1.114759	0.040*	0.089 (3)

### Tris(naphthalen-2-yl isocyanide)(η4-tetraphenylcyclopentadienone)iron acetone hemisolvate (twin) Atomic displacement parameters (Å^2^)

**Table:**

	*U*^11^	*U*^22^	*U*^33^	*U*^12^	*U*^13^	*U*^23^
Fe1A	0.01549 (19)	0.0134 (2)	0.01623 (19)	−0.00060 (16)	0.00133 (14)	0.00003 (16)
O1A	0.0235 (10)	0.0133 (10)	0.0222 (10)	−0.0006 (8)	0.0002 (8)	−0.0005 (8)
N1A	0.0236 (12)	0.0205 (13)	0.0219 (12)	−0.0004 (10)	−0.0004 (9)	0.0023 (10)
N2A	0.0257 (12)	0.0216 (13)	0.0264 (13)	−0.0041 (10)	0.0068 (10)	−0.0062 (10)
N3A	0.0202 (12)	0.0248 (13)	0.0250 (12)	−0.0038 (10)	0.0026 (10)	0.0022 (10)
C1A	0.0190 (12)	0.0185 (14)	0.0168 (12)	−0.0016 (10)	0.0016 (10)	−0.0001 (10)
C2A	0.0164 (12)	0.0166 (13)	0.0223 (14)	−0.0021 (10)	0.0003 (10)	0.0002 (10)
C3A	0.0221 (13)	0.0143 (13)	0.0200 (13)	0.0004 (10)	0.0008 (10)	−0.0008 (10)
C4A	0.0190 (13)	0.0210 (15)	0.0173 (13)	−0.0013 (11)	−0.0004 (10)	0.0004 (10)
C5A	0.0171 (12)	0.0166 (14)	0.0167 (12)	−0.0010 (10)	−0.0007 (10)	−0.0007 (10)
C6A	0.0145 (11)	0.0189 (14)	0.0159 (12)	−0.0017 (10)	−0.0022 (9)	−0.0005 (10)
C7A	0.0161 (12)	0.0164 (14)	0.0175 (12)	−0.0012 (10)	−0.0016 (9)	−0.0015 (10)
C8A	0.0164 (12)	0.0160 (13)	0.0175 (12)	−0.0026 (10)	−0.0023 (10)	−0.0014 (10)
C9A	0.0220 (13)	0.0186 (14)	0.0194 (13)	0.0019 (11)	0.0045 (10)	0.0031 (11)
C10A	0.0215 (14)	0.0264 (16)	0.0226 (14)	−0.0013 (12)	−0.0015 (11)	−0.0009 (12)
C11A	0.0252 (14)	0.0194 (15)	0.0288 (16)	−0.0034 (11)	0.0016 (12)	−0.0035 (12)
C12A	0.0239 (14)	0.0200 (15)	0.0221 (14)	0.0010 (11)	0.0065 (11)	0.0003 (11)
C13A	0.0380 (18)	0.0167 (15)	0.0353 (17)	0.0011 (13)	0.0021 (14)	−0.0014 (13)
C14A	0.0407 (19)	0.0238 (17)	0.0363 (18)	0.0106 (14)	0.0040 (15)	0.0079 (14)
C15A	0.0254 (14)	0.0333 (18)	0.0245 (15)	0.0109 (13)	−0.0008 (12)	0.0024 (13)
C16A	0.0211 (13)	0.0254 (16)	0.0230 (14)	0.0028 (11)	0.0016 (11)	−0.0015 (11)
C17A	0.0181 (13)	0.0199 (15)	0.0198 (13)	0.0027 (10)	0.0054 (10)	−0.0009 (11)
C18A	0.0196 (13)	0.0182 (14)	0.0204 (13)	−0.0014 (11)	0.0021 (10)	−0.0009 (11)
C19A	0.0222 (13)	0.0191 (14)	0.0226 (14)	−0.0042 (11)	0.0069 (11)	−0.0038 (11)
C20A	0.0195 (13)	0.0214 (15)	0.0277 (15)	0.0021 (11)	0.0017 (11)	−0.0021 (12)
C21A	0.0281 (15)	0.0227 (15)	0.0226 (14)	0.0005 (12)	0.0020 (11)	0.0000 (11)
C22A	0.0297 (15)	0.0167 (14)	0.0259 (15)	0.0011 (11)	0.0091 (12)	0.0024 (11)
C23A	0.047 (2)	0.0261 (17)	0.0285 (16)	0.0037 (14)	0.0164 (15)	0.0036 (13)
C24A	0.053 (2)	0.037 (2)	0.048 (2)	0.0112 (17)	0.0338 (19)	0.0068 (17)
C25A	0.0354 (19)	0.052 (3)	0.061 (3)	0.0202 (17)	0.0257 (18)	0.016 (2)
C26A	0.0270 (16)	0.045 (2)	0.0407 (19)	0.0096 (15)	0.0107 (14)	0.0114 (16)
C27A	0.0230 (14)	0.0229 (15)	0.0278 (15)	0.0012 (11)	0.0069 (11)	0.0064 (12)
C28A	0.0219 (13)	0.0266 (16)	0.0219 (14)	−0.0024 (11)	0.0014 (11)	0.0026 (12)
C29A	0.0178 (13)	0.0239 (16)	0.0233 (14)	−0.0059 (11)	0.0025 (11)	0.0029 (11)
C30A	0.0261 (14)	0.0229 (16)	0.0279 (15)	0.0000 (12)	0.0026 (12)	−0.0013 (12)
C31A	0.0330 (16)	0.0208 (16)	0.0306 (16)	−0.0076 (12)	−0.0033 (13)	0.0019 (12)
C32A	0.0242 (14)	0.0340 (18)	0.0218 (14)	−0.0094 (12)	−0.0014 (11)	0.0064 (12)
C33A	0.0339 (18)	0.048 (2)	0.0344 (18)	−0.0175 (16)	−0.0028 (14)	0.0141 (16)
C34A	0.0286 (18)	0.086 (4)	0.0316 (19)	−0.0132 (19)	0.0073 (15)	0.018 (2)
C35A	0.0292 (18)	0.078 (3)	0.0332 (19)	0.0036 (18)	0.0103 (15)	0.0026 (19)
C36A	0.0261 (16)	0.049 (2)	0.0303 (17)	0.0019 (15)	0.0051 (13)	−0.0031 (16)
C37A	0.0180 (13)	0.0327 (18)	0.0216 (14)	−0.0028 (11)	−0.0012 (11)	0.0023 (12)
C38A	0.0217 (13)	0.0205 (15)	0.0262 (15)	−0.0013 (11)	−0.0005 (11)	0.0019 (12)
C39A	0.0192 (13)	0.0227 (15)	0.0150 (12)	0.0030 (11)	−0.0004 (10)	0.0020 (11)
C40A	0.0219 (13)	0.0191 (14)	0.0206 (13)	0.0019 (11)	0.0019 (11)	0.0004 (11)
C41A	0.0235 (14)	0.0311 (17)	0.0236 (14)	0.0031 (12)	0.0050 (11)	0.0033 (12)
C42A	0.0283 (16)	0.0359 (19)	0.0223 (14)	0.0063 (13)	0.0066 (12)	−0.0029 (13)
C43A	0.0344 (16)	0.0239 (16)	0.0269 (15)	0.0057 (13)	0.0053 (13)	−0.0054 (12)
C44A	0.0244 (14)	0.0218 (15)	0.0238 (14)	0.0015 (11)	0.0027 (11)	−0.0014 (11)
C45A	0.0172 (12)	0.0194 (14)	0.0230 (14)	0.0017 (10)	0.0041 (10)	0.0025 (11)
C46A	0.0193 (13)	0.0215 (15)	0.0281 (15)	−0.0007 (11)	0.0036 (11)	0.0027 (12)
C47A	0.0262 (15)	0.0192 (15)	0.0399 (18)	−0.0007 (12)	0.0036 (13)	0.0074 (13)
C48A	0.0285 (15)	0.0285 (18)	0.0359 (17)	0.0013 (13)	0.0079 (13)	0.0161 (14)
C49A	0.0283 (15)	0.0314 (17)	0.0239 (15)	0.0063 (13)	0.0041 (12)	0.0043 (13)
C50A	0.0227 (13)	0.0214 (15)	0.0231 (14)	0.0024 (11)	0.0035 (11)	0.0011 (11)
C51A	0.0205 (13)	0.0174 (14)	0.0167 (12)	0.0010 (10)	0.0034 (10)	0.0003 (10)
C52A	0.0224 (13)	0.0206 (14)	0.0215 (14)	−0.0003 (11)	0.0022 (11)	−0.0017 (11)
C53A	0.0204 (14)	0.0339 (18)	0.0276 (15)	0.0047 (12)	0.0044 (11)	0.0029 (13)
C54A	0.0312 (16)	0.0301 (18)	0.0317 (17)	0.0142 (13)	0.0111 (13)	0.0066 (14)
C55A	0.0386 (17)	0.0159 (15)	0.0301 (16)	0.0035 (12)	0.0076 (13)	0.0007 (12)
C56A	0.0241 (14)	0.0185 (15)	0.0247 (14)	−0.0017 (11)	0.0037 (11)	−0.0018 (11)
C57A	0.0159 (12)	0.0200 (14)	0.0197 (13)	0.0007 (10)	−0.0008 (10)	0.0024 (11)
C58A	0.0205 (13)	0.0203 (15)	0.0251 (14)	−0.0030 (11)	0.0027 (11)	0.0006 (11)
C59A	0.0246 (14)	0.0250 (16)	0.0326 (16)	−0.0049 (12)	0.0019 (12)	0.0076 (13)
C60A	0.0228 (15)	0.0354 (19)	0.0285 (16)	−0.0013 (13)	0.0059 (12)	0.0103 (14)
C61A	0.0232 (14)	0.0335 (18)	0.0231 (14)	0.0034 (12)	0.0043 (12)	0.0024 (13)
C62A	0.0205 (13)	0.0221 (15)	0.0217 (14)	−0.0009 (11)	0.0016 (11)	0.0014 (11)
Fe1B	0.01538 (19)	0.0150 (2)	0.0168 (2)	0.00048 (16)	0.00164 (15)	0.00101 (16)
O1B	0.0217 (10)	0.0164 (11)	0.0242 (10)	−0.0007 (7)	−0.0012 (8)	0.0019 (8)
N1B	0.0182 (11)	0.0223 (13)	0.0266 (13)	0.0014 (9)	0.0024 (9)	0.0048 (10)
N2B	0.0259 (12)	0.0201 (13)	0.0303 (13)	−0.0019 (10)	0.0082 (10)	−0.0017 (10)
N3B	0.0242 (12)	0.0288 (14)	0.0182 (12)	0.0023 (10)	0.0017 (9)	−0.0013 (10)
C1B	0.0148 (12)	0.0186 (14)	0.0226 (14)	−0.0012 (10)	0.0015 (10)	−0.0015 (11)
C2B	0.0217 (13)	0.0168 (14)	0.0199 (13)	0.0024 (10)	0.0019 (10)	0.0011 (10)
C3B	0.0158 (12)	0.0190 (14)	0.0231 (13)	0.0006 (10)	−0.0009 (10)	0.0022 (11)
C4B	0.0163 (12)	0.0216 (16)	0.0190 (13)	0.0002 (10)	−0.0009 (10)	0.0020 (11)
C5B	0.0146 (12)	0.0179 (14)	0.0215 (13)	−0.0003 (10)	−0.0006 (10)	0.0004 (11)
C6B	0.0163 (12)	0.0190 (14)	0.0193 (12)	0.0007 (10)	−0.0007 (10)	0.0001 (11)
C7B	0.0168 (12)	0.0186 (15)	0.0167 (12)	0.0006 (10)	−0.0014 (9)	−0.0001 (10)
C8B	0.0190 (12)	0.0178 (14)	0.0188 (13)	−0.0010 (10)	0.0009 (10)	−0.0012 (11)
C9B	0.0195 (13)	0.0205 (15)	0.0225 (14)	0.0037 (11)	0.0033 (11)	0.0034 (11)
C10B	0.0199 (14)	0.0262 (16)	0.0249 (15)	0.0003 (11)	0.0006 (11)	0.0019 (12)
C11B	0.0210 (14)	0.0271 (16)	0.0234 (14)	0.0038 (12)	−0.0002 (11)	0.0049 (12)
C12B	0.0255 (14)	0.0239 (16)	0.0215 (14)	0.0054 (12)	0.0050 (11)	0.0027 (11)
C13B	0.0344 (16)	0.0255 (17)	0.0234 (15)	0.0079 (13)	0.0010 (12)	0.0053 (12)
C14B	0.0471 (19)	0.0192 (16)	0.0274 (16)	0.0036 (13)	0.0050 (14)	0.0048 (12)
C15B	0.0394 (17)	0.0239 (16)	0.0280 (16)	−0.0026 (13)	0.0038 (13)	−0.0017 (13)
C16B	0.0282 (15)	0.0261 (17)	0.0248 (15)	0.0015 (12)	0.0018 (12)	0.0017 (12)
C17B	0.0217 (13)	0.0210 (15)	0.0215 (14)	0.0036 (11)	0.0045 (11)	0.0025 (11)
C18B	0.0187 (13)	0.0248 (16)	0.0215 (14)	0.0038 (11)	0.0017 (11)	0.0026 (11)
C19B	0.0196 (16)	0.0173 (17)	0.0304 (19)	−0.0010 (13)	0.0069 (14)	0.0008 (15)
C20B	0.0245 (16)	0.0259 (18)	0.0241 (17)	−0.0002 (13)	0.0043 (13)	0.0024 (14)
C21B	0.0210 (15)	0.0283 (18)	0.0307 (17)	−0.0009 (13)	0.0007 (13)	−0.0017 (14)
C22B	0.0205 (16)	0.0205 (17)	0.0347 (17)	0.0026 (13)	0.0031 (13)	0.0039 (14)
C23B	0.0197 (16)	0.0295 (19)	0.041 (2)	0.0018 (14)	0.0039 (15)	0.0076 (17)
C24B	0.0253 (16)	0.0265 (18)	0.053 (2)	0.0005 (13)	0.0127 (17)	0.0136 (19)
C25B	0.037 (2)	0.035 (2)	0.041 (2)	0.0037 (16)	0.0168 (17)	0.0135 (18)
C26B	0.0345 (18)	0.035 (2)	0.0312 (19)	0.0017 (15)	0.0074 (15)	0.0055 (16)
C27B	0.0223 (17)	0.022 (2)	0.0308 (18)	0.0012 (14)	0.0062 (14)	0.0021 (14)
C28B	0.0225 (15)	0.0247 (17)	0.0246 (16)	−0.0008 (13)	0.0009 (12)	−0.0007 (13)
C29B	0.0244 (17)	0.0226 (19)	0.0180 (16)	0.0042 (14)	0.0042 (13)	0.0024 (14)
C30B	0.0213 (16)	0.0259 (17)	0.0216 (15)	0.0003 (13)	0.0033 (13)	−0.0005 (12)
C31B	0.0239 (17)	0.0272 (18)	0.0195 (15)	0.0008 (13)	−0.0002 (13)	−0.0005 (13)
C32B	0.0237 (16)	0.0239 (17)	0.0186 (15)	0.0044 (13)	0.0013 (13)	0.0051 (13)
C33B	0.0341 (18)	0.0293 (19)	0.0204 (16)	0.0072 (14)	0.0064 (13)	0.0031 (13)
C34B	0.0369 (19)	0.040 (2)	0.0251 (18)	0.0136 (16)	0.0144 (15)	0.0086 (15)
C35B	0.0250 (17)	0.043 (2)	0.0357 (19)	0.0058 (15)	0.0117 (14)	0.0155 (16)
C36B	0.0253 (17)	0.037 (2)	0.0305 (18)	−0.0027 (14)	0.0041 (14)	0.0079 (15)
C37B	0.0196 (15)	0.024 (2)	0.0234 (19)	0.0030 (13)	0.0026 (14)	0.0056 (15)
C38B	0.0239 (16)	0.027 (2)	0.0215 (16)	−0.0025 (14)	0.0002 (13)	−0.0002 (14)
C39B	0.0180 (13)	0.0201 (15)	0.0240 (14)	−0.0018 (10)	0.0030 (11)	0.0013 (11)
C40B	0.0225 (15)	0.0282 (17)	0.047 (2)	0.0053 (13)	0.0099 (14)	0.0117 (15)
C41B	0.0297 (17)	0.041 (2)	0.061 (3)	0.0050 (15)	0.0252 (17)	0.0108 (18)
C42B	0.0408 (19)	0.041 (2)	0.042 (2)	−0.0020 (16)	0.0232 (16)	0.0099 (16)
C43B	0.0313 (17)	0.047 (2)	0.0320 (17)	0.0013 (15)	0.0077 (14)	0.0160 (16)
C44B	0.0239 (14)	0.0328 (18)	0.0301 (16)	0.0023 (13)	0.0072 (12)	0.0105 (13)
C45B	0.0180 (12)	0.0180 (14)	0.0208 (13)	0.0011 (10)	0.0042 (10)	0.0042 (11)
C46B	0.0198 (13)	0.0242 (15)	0.0255 (15)	0.0027 (11)	0.0007 (11)	−0.0017 (12)
C47B	0.0260 (15)	0.0248 (16)	0.0288 (16)	0.0035 (12)	0.0041 (12)	−0.0046 (12)
C48B	0.0242 (14)	0.0226 (16)	0.0318 (16)	0.0057 (11)	0.0067 (12)	0.0027 (12)
C49B	0.0240 (15)	0.0319 (18)	0.0320 (16)	0.0087 (13)	−0.0070 (13)	−0.0027 (14)
C50B	0.0273 (15)	0.0246 (16)	0.0283 (15)	0.0062 (12)	−0.0046 (12)	−0.0068 (12)
C51B	0.0168 (12)	0.0213 (14)	0.0188 (13)	0.0016 (10)	0.0016 (10)	0.0028 (11)
C52B	0.0274 (14)	0.0196 (15)	0.0226 (14)	0.0047 (11)	0.0008 (11)	−0.0005 (11)
C53B	0.0327 (16)	0.0298 (17)	0.0193 (14)	0.0044 (13)	0.0028 (12)	0.0033 (12)
C54B	0.0317 (16)	0.0254 (17)	0.0259 (15)	0.0020 (12)	0.0085 (12)	0.0086 (12)
C55B	0.0290 (15)	0.0189 (15)	0.0288 (15)	−0.0029 (11)	0.0073 (12)	0.0007 (12)
C56B	0.0259 (14)	0.0223 (15)	0.0217 (14)	−0.0038 (11)	0.0056 (11)	−0.0003 (12)
C57B	0.0207 (13)	0.0196 (14)	0.0200 (13)	0.0046 (11)	0.0018 (10)	0.0035 (11)
C58B	0.0262 (14)	0.0205 (15)	0.0304 (15)	−0.0006 (11)	0.0072 (12)	−0.0019 (12)
C59B	0.0343 (16)	0.0242 (17)	0.0297 (16)	−0.0008 (13)	0.0073 (13)	−0.0076 (13)
C60B	0.0278 (15)	0.0309 (17)	0.0256 (15)	0.0062 (13)	0.0083 (12)	−0.0007 (13)
C61B	0.0229 (14)	0.0313 (17)	0.0281 (15)	0.0011 (12)	0.0067 (12)	0.0030 (13)
C62B	0.0218 (13)	0.0236 (16)	0.0226 (14)	0.0005 (11)	0.0014 (11)	−0.0010 (11)
O1	0.0399 (14)	0.0360 (14)	0.0473 (14)	−0.0044 (11)	0.0060 (11)	−0.0055 (12)
C1	0.0410 (19)	0.0263 (18)	0.0365 (18)	−0.0007 (14)	0.0075 (14)	−0.0054 (14)
C2	0.048 (2)	0.088 (4)	0.038 (2)	−0.016 (2)	0.0090 (18)	−0.012 (2)
C3	0.053 (2)	0.038 (2)	0.0365 (19)	−0.0081 (17)	0.0046 (16)	−0.0040 (16)
C29C	0.024 (10)	0.029 (15)	0.024 (11)	−0.001 (9)	0.004 (8)	−0.002 (9)
C38C	0.0213 (16)	0.0259 (17)	0.0216 (15)	0.0003 (13)	0.0033 (13)	−0.0005 (12)
C37C	0.021 (10)	0.020 (11)	0.019 (10)	0.003 (8)	0.000 (8)	0.003 (8)
C36C	0.031 (11)	0.010 (11)	0.021 (10)	0.008 (8)	0.004 (8)	0.001 (8)
C35C	0.0341 (18)	0.0293 (19)	0.0204 (16)	0.0072 (14)	0.0064 (13)	0.0031 (13)
C34C	0.0341 (18)	0.0293 (19)	0.0204 (16)	0.0072 (14)	0.0064 (13)	0.0031 (13)
C33C	0.031 (11)	0.034 (12)	0.011 (10)	0.012 (8)	0.002 (8)	0.003 (8)
C32C	0.025 (10)	0.025 (12)	0.013 (12)	0.001 (9)	0.003 (9)	0.001 (9)
C31C	0.030 (12)	0.019 (13)	0.022 (11)	−0.001 (10)	0.008 (9)	0.000 (9)
C30C	0.021 (10)	0.026 (15)	0.021 (12)	0.004 (9)	0.005 (9)	0.000 (10)
C19C	0.025 (11)	0.014 (12)	0.026 (8)	0.001 (10)	0.004 (7)	−0.006 (9)
C27C	0.030 (11)	0.033 (14)	0.043 (10)	−0.007 (10)	0.008 (8)	−0.004 (10)
C25C	0.025 (12)	0.032 (16)	0.055 (9)	0.000 (11)	0.013 (10)	0.014 (12)
C20C	0.0225 (15)	0.0247 (17)	0.0246 (16)	−0.0008 (13)	0.0009 (12)	−0.0007 (13)
C24C	0.0253 (16)	0.0265 (18)	0.053 (2)	0.0005 (13)	0.0127 (17)	0.0136 (19)
C21C	0.017 (9)	0.017 (13)	0.047 (11)	−0.001 (8)	0.005 (8)	0.000 (11)
C26C	0.0205 (16)	0.0205 (17)	0.0347 (17)	0.0026 (13)	0.0031 (13)	0.0039 (14)
C22C	0.012 (10)	0.029 (16)	0.045 (10)	0.001 (9)	0.007 (8)	0.002 (11)
C28C	0.025 (10)	0.024 (13)	0.025 (11)	−0.006 (9)	0.002 (9)	−0.011 (11)
C23C	0.018 (10)	0.034 (14)	0.052 (12)	0.005 (9)	0.014 (8)	0.016 (12)

### Tris(naphthalen-2-yl isocyanide)(η4-tetraphenylcyclopentadienone)iron acetone hemisolvate (twin) Geometric parameters (Å, º)

**Table:**

Fe1A—C1A	1.835 (3)	C7B—C51B	1.480 (4)
Fe1A—C2A	1.829 (3)	C8B—C57B	1.484 (4)
Fe1A—C3A	1.828 (3)	C9B—C10B	1.414 (4)
Fe1A—C4A	2.367 (3)	C9B—C18B	1.373 (4)
Fe1A—C5A	2.142 (3)	C10B—H10B	0.9500
Fe1A—C6A	2.070 (3)	C10B—C11B	1.367 (5)
Fe1A—C7A	2.067 (3)	C11B—H11B	0.9500
Fe1A—C8A	2.141 (3)	C11B—C12B	1.423 (5)
O1A—C4A	1.247 (4)	C12B—C13B	1.411 (4)
N1A—C1A	1.160 (4)	C12B—C17B	1.428 (4)
N1A—C9A	1.399 (4)	C13B—H13B	0.9500
N2A—C2A	1.165 (4)	C13B—C14B	1.364 (5)
N2A—C19A	1.396 (4)	C14B—H14B	0.9500
N3A—C3A	1.167 (4)	C14B—C15B	1.413 (5)
N3A—C29A	1.403 (4)	C15B—H15B	0.9500
C4A—C5A	1.480 (4)	C15B—C16B	1.364 (5)
C4A—C8A	1.476 (4)	C16B—H16B	0.9500
C5A—C6A	1.438 (4)	C16B—C17B	1.419 (5)
C5A—C39A	1.488 (4)	C17B—C18B	1.421 (4)
C6A—C7A	1.442 (4)	C18B—H18B	0.9500
C6A—C45A	1.486 (4)	C19B—C20B	1.413 (5)
C7A—C8A	1.447 (4)	C19B—C28B	1.377 (5)
C7A—C51A	1.484 (4)	C20B—H20B	0.9500
C8A—C57A	1.481 (4)	C20B—C21B	1.367 (5)
C9A—C10A	1.412 (4)	C21B—H21B	0.9500
C9A—C18A	1.372 (4)	C21B—C22B	1.416 (5)
C10A—H10A	0.9500	C22B—C23B	1.424 (5)
C10A—C11A	1.361 (5)	C22B—C27B	1.417 (5)
C11A—H11A	0.9500	C23B—H23B	0.9500
C11A—C12A	1.425 (4)	C23B—C24B	1.371 (6)
C12A—C13A	1.419 (4)	C24B—H24B	0.9500
C12A—C17A	1.416 (4)	C24B—C25B	1.409 (6)
C13A—H13A	0.9500	C25B—H25B	0.9500
C13A—C14A	1.366 (5)	C25B—C26B	1.369 (5)
C14A—H14A	0.9500	C26B—H26B	0.9500
C14A—C15A	1.412 (5)	C26B—C27B	1.415 (5)
C15A—H15A	0.9500	C27B—C28B	1.419 (5)
C15A—C16A	1.368 (5)	C28B—H28B	0.9500
C16A—H16A	0.9500	C29B—C30B	1.416 (5)
C16A—C17A	1.420 (4)	C29B—C38B	1.370 (5)
C17A—C18A	1.413 (4)	C30B—H30B	0.9500
C18A—H18A	0.9500	C30B—C31B	1.370 (5)
C19A—C20A	1.413 (4)	C31B—H31B	0.9500
C19A—C28A	1.369 (4)	C31B—C32B	1.416 (5)
C20A—H20A	0.9500	C32B—C33B	1.419 (5)
C20A—C21A	1.366 (4)	C32B—C37B	1.420 (5)
C21A—H21A	0.9500	C33B—H33B	0.9500
C21A—C22A	1.417 (4)	C33B—C34B	1.370 (5)
C22A—C23A	1.425 (4)	C34B—H34B	0.9500
C22A—C27A	1.414 (4)	C34B—C35B	1.401 (6)
C23A—H23A	0.9500	C35B—H35B	0.9500
C23A—C24A	1.366 (5)	C35B—C36B	1.367 (5)
C24A—H24A	0.9500	C36B—H36B	0.9500
C24A—C25A	1.402 (7)	C36B—C37B	1.418 (5)
C25A—H25A	0.9500	C37B—C38B	1.419 (5)
C25A—C26A	1.375 (5)	C38B—H38B	0.9500
C26A—H26A	0.9500	C39B—C40B	1.404 (4)
C26A—C27A	1.420 (4)	C39B—C44B	1.398 (4)
C27A—C28A	1.416 (4)	C40B—H40B	0.9500
C28A—H28A	0.9500	C40B—C41B	1.388 (5)
C29A—C30A	1.415 (5)	C41B—H41B	0.9500
C29A—C38A	1.369 (4)	C41B—C42B	1.385 (6)
C30A—H30A	0.9500	C42B—H42B	0.9500
C30A—C31A	1.372 (5)	C42B—C43B	1.385 (5)
C31A—H31A	0.9500	C43B—H43B	0.9500
C31A—C32A	1.416 (5)	C43B—C44B	1.391 (4)
C32A—C33A	1.425 (4)	C44B—H44B	0.9500
C32A—C37A	1.425 (5)	C45B—C46B	1.389 (4)
C33A—H33A	0.9500	C45B—C50B	1.399 (4)
C33A—C34A	1.365 (6)	C46B—H46B	0.9500
C34A—H34A	0.9500	C46B—C47B	1.394 (4)
C34A—C35A	1.413 (7)	C47B—H47B	0.9500
C35A—H35A	0.9500	C47B—C48B	1.380 (5)
C35A—C36A	1.358 (5)	C48B—H48B	0.9500
C36A—H36A	0.9500	C48B—C49B	1.379 (5)
C36A—C37A	1.416 (5)	C49B—H49B	0.9500
C37A—C38A	1.408 (4)	C49B—C50B	1.387 (4)
C38A—H38A	0.9500	C50B—H50B	0.9500
C39A—C40A	1.400 (4)	C51B—C52B	1.400 (4)
C39A—C44A	1.400 (4)	C51B—C56B	1.390 (4)
C40A—H40A	0.9500	C52B—H52B	0.9500
C40A—C41A	1.393 (4)	C52B—C53B	1.394 (4)
C41A—H41A	0.9500	C53B—H53B	0.9500
C41A—C42A	1.391 (5)	C53B—C54B	1.380 (5)
C42A—H42A	0.9500	C54B—H54B	0.9500
C42A—C43A	1.386 (5)	C54B—C55B	1.392 (5)
C43A—H43A	0.9500	C55B—H55B	0.9500
C43A—C44A	1.388 (4)	C55B—C56B	1.386 (4)
C44A—H44A	0.9500	C56B—H56B	0.9500
C45A—C46A	1.395 (4)	C57B—C58B	1.393 (4)
C45A—C50A	1.398 (4)	C57B—C62B	1.398 (4)
C46A—H46A	0.9500	C58B—H58B	0.9500
C46A—C47A	1.394 (4)	C58B—C59B	1.387 (4)
C47A—H47A	0.9500	C59B—H59B	0.9500
C47A—C48A	1.386 (5)	C59B—C60B	1.389 (5)
C48A—H48A	0.9500	C60B—H60B	0.9500
C48A—C49A	1.385 (5)	C60B—C61B	1.391 (5)
C49A—H49A	0.9500	C61B—H61B	0.9500
C49A—C50A	1.390 (4)	C61B—C62B	1.390 (4)
C50A—H50A	0.9500	C62B—H62B	0.9500
C51A—C52A	1.396 (4)	O1—C1	1.211 (4)
C51A—C56A	1.394 (4)	C1—C2	1.502 (5)
C52A—H52A	0.9500	C1—C3	1.495 (5)
C52A—C53A	1.396 (4)	C2—H2A	0.9800
C53A—H53A	0.9500	C2—H2B	0.9800
C53A—C54A	1.384 (5)	C2—H2C	0.9800
C54A—H54A	0.9500	C3—H3A	0.9800
C54A—C55A	1.379 (5)	C3—H3B	0.9800
C55A—H55A	0.9500	C3—H3C	0.9800
C55A—C56A	1.396 (4)	C29C—C38C	1.39 (2)
C56A—H56A	0.9500	C29C—C30C	1.391 (19)
C57A—C58A	1.405 (4)	C38C—H38C	0.9500
C57A—C62A	1.402 (4)	C38C—C37C	1.419 (19)
C58A—H58A	0.9500	C37C—C36C	1.403 (19)
C58A—C59A	1.392 (4)	C37C—C32C	1.410 (19)
C59A—H59A	0.9500	C36C—H36C	0.9500
C59A—C60A	1.384 (5)	C36C—C35C	1.408 (19)
C60A—H60A	0.9500	C35C—H35C	0.9500
C60A—C61A	1.392 (5)	C35C—C34C	1.409 (19)
C61A—H61A	0.9500	C34C—H34C	0.9500
C61A—C62A	1.385 (4)	C34C—C33C	1.39 (2)
C62A—H62A	0.9500	C33C—H33C	0.9500
Fe1B—C1B	1.836 (3)	C33C—C32C	1.423 (19)
Fe1B—C2B	1.840 (3)	C32C—C31C	1.414 (19)
Fe1B—C3B	1.823 (3)	C31C—H31C	0.9500
Fe1B—C4B	2.373 (3)	C31C—C30C	1.398 (19)
Fe1B—C5B	2.116 (3)	C30C—H30C	0.9500
Fe1B—C6B	2.070 (3)	C19C—C20C	1.385 (19)
Fe1B—C7B	2.085 (3)	C19C—C28C	1.392 (19)
Fe1B—C8B	2.142 (3)	C27C—C26C	1.402 (18)
O1B—C4B	1.243 (4)	C27C—C22C	1.404 (19)
N1B—C1B	1.163 (4)	C27C—C28C	1.403 (19)
N1B—C9B	1.397 (4)	C25C—H25C	0.9500
N2B—C2B	1.172 (4)	C25C—C24C	1.39 (2)
N2B—C19B	1.410 (4)	C25C—C26C	1.391 (19)
N2B—C19C	1.39 (2)	C20C—H20C	0.9500
N3B—C3B	1.172 (4)	C20C—C21C	1.394 (19)
N3B—C29B	1.404 (4)	C24C—H24C	0.9500
N3B—C29C	1.38 (2)	C24C—C23C	1.39 (2)
C4B—C5B	1.481 (4)	C21C—H21C	0.9500
C4B—C8B	1.477 (4)	C21C—C22C	1.404 (19)
C5B—C6B	1.442 (4)	C26C—H26C	0.9500
C5B—C39B	1.479 (4)	C22C—C23C	1.400 (18)
C6B—C7B	1.441 (4)	C28C—H28C	0.9500
C6B—C45B	1.491 (4)	C23C—H23C	0.9500
C7B—C8B	1.436 (4)		
			
C1A—Fe1A—C4A	79.78 (11)	C5B—C4B—Fe1B	61.55 (15)
C1A—Fe1A—C5A	98.52 (12)	C8B—C4B—Fe1B	62.62 (15)
C1A—Fe1A—C6A	138.31 (12)	C8B—C4B—C5B	104.3 (2)
C1A—Fe1A—C7A	137.35 (12)	C4B—C5B—Fe1B	80.46 (16)
C1A—Fe1A—C8A	97.18 (12)	C6B—C5B—Fe1B	68.15 (15)
C2A—Fe1A—C1A	95.62 (13)	C6B—C5B—C4B	108.6 (2)
C2A—Fe1A—C4A	132.89 (11)	C6B—C5B—C39B	127.7 (3)
C2A—Fe1A—C5A	159.93 (11)	C39B—C5B—Fe1B	126.0 (2)
C2A—Fe1A—C6A	123.96 (12)	C39B—C5B—C4B	122.8 (3)
C2A—Fe1A—C7A	92.63 (12)	C5B—C6B—Fe1B	71.57 (15)
C2A—Fe1A—C8A	98.10 (11)	C5B—C6B—C45B	128.9 (2)
C3A—Fe1A—C1A	97.51 (12)	C7B—C6B—Fe1B	70.28 (14)
C3A—Fe1A—C2A	95.24 (12)	C7B—C6B—C5B	107.6 (2)
C3A—Fe1A—C4A	131.86 (11)	C7B—C6B—C45B	122.4 (2)
C3A—Fe1A—C5A	96.94 (12)	C45B—C6B—Fe1B	133.1 (2)
C3A—Fe1A—C6A	91.83 (12)	C6B—C7B—Fe1B	69.13 (14)
C3A—Fe1A—C7A	123.31 (12)	C6B—C7B—C51B	123.2 (3)
C3A—Fe1A—C8A	159.05 (12)	C8B—C7B—Fe1B	72.29 (15)
C5A—Fe1A—C4A	37.90 (10)	C8B—C7B—C6B	108.6 (2)
C6A—Fe1A—C4A	64.25 (10)	C8B—C7B—C51B	127.4 (2)
C6A—Fe1A—C5A	39.87 (11)	C51B—C7B—Fe1B	132.2 (2)
C6A—Fe1A—C8A	67.30 (10)	C4B—C8B—Fe1B	79.63 (17)
C7A—Fe1A—C4A	64.44 (10)	C4B—C8B—C57B	125.0 (3)
C7A—Fe1A—C5A	67.34 (10)	C7B—C8B—Fe1B	68.01 (15)
C7A—Fe1A—C6A	40.80 (10)	C7B—C8B—C4B	108.3 (2)
C7A—Fe1A—C8A	40.18 (11)	C7B—C8B—C57B	124.6 (3)
C8A—Fe1A—C4A	37.80 (10)	C57B—C8B—Fe1B	131.2 (2)
C8A—Fe1A—C5A	66.10 (10)	N1B—C9B—C10B	119.0 (3)
C1A—N1A—C9A	168.6 (3)	C18B—C9B—N1B	118.8 (3)
C2A—N2A—C19A	170.5 (3)	C18B—C9B—C10B	122.1 (3)
C3A—N3A—C29A	168.5 (3)	C9B—C10B—H10B	120.3
N1A—C1A—Fe1A	175.5 (3)	C11B—C10B—C9B	119.4 (3)
N2A—C2A—Fe1A	176.5 (3)	C11B—C10B—H10B	120.3
N3A—C3A—Fe1A	175.5 (3)	C10B—C11B—H11B	119.5
O1A—C4A—Fe1A	133.9 (2)	C10B—C11B—C12B	120.9 (3)
O1A—C4A—C5A	128.0 (3)	C12B—C11B—H11B	119.5
O1A—C4A—C8A	127.3 (3)	C11B—C12B—C17B	118.8 (3)
C5A—C4A—Fe1A	62.77 (15)	C13B—C12B—C11B	122.5 (3)
C8A—C4A—Fe1A	62.76 (14)	C13B—C12B—C17B	118.7 (3)
C8A—C4A—C5A	104.4 (2)	C12B—C13B—H13B	119.5
C4A—C5A—Fe1A	79.34 (16)	C14B—C13B—C12B	121.0 (3)
C4A—C5A—C39A	123.8 (3)	C14B—C13B—H13B	119.5
C6A—C5A—Fe1A	67.36 (15)	C13B—C14B—H14B	119.8
C6A—C5A—C4A	108.8 (2)	C13B—C14B—C15B	120.3 (3)
C6A—C5A—C39A	125.6 (2)	C15B—C14B—H14B	119.8
C39A—C5A—Fe1A	131.10 (19)	C14B—C15B—H15B	119.8
C5A—C6A—Fe1A	72.76 (15)	C16B—C15B—C14B	120.5 (3)
C5A—C6A—C7A	108.3 (2)	C16B—C15B—H15B	119.8
C5A—C6A—C45A	125.3 (2)	C15B—C16B—H16B	119.8
C7A—C6A—Fe1A	69.51 (15)	C15B—C16B—C17B	120.4 (3)
C7A—C6A—C45A	125.3 (2)	C17B—C16B—H16B	119.8
C45A—C6A—Fe1A	132.87 (19)	C16B—C17B—C12B	119.0 (3)
C6A—C7A—Fe1A	69.69 (14)	C16B—C17B—C18B	121.3 (3)
C6A—C7A—C8A	107.8 (2)	C18B—C17B—C12B	119.6 (3)
C6A—C7A—C51A	124.5 (2)	C9B—C18B—C17B	119.1 (3)
C8A—C7A—Fe1A	72.65 (15)	C9B—C18B—H18B	120.5
C8A—C7A—C51A	126.3 (2)	C17B—C18B—H18B	120.5
C51A—C7A—Fe1A	133.59 (19)	N2B—C19B—C20B	119.0 (3)
C4A—C8A—Fe1A	79.43 (16)	C28B—C19B—N2B	119.4 (3)
C4A—C8A—C57A	124.0 (3)	C28B—C19B—C20B	121.6 (3)
C7A—C8A—Fe1A	67.17 (15)	C19B—C20B—H20B	120.3
C7A—C8A—C4A	108.9 (2)	C21B—C20B—C19B	119.3 (3)
C7A—C8A—C57A	125.5 (2)	C21B—C20B—H20B	120.3
C57A—C8A—Fe1A	130.93 (19)	C20B—C21B—H21B	119.5
N1A—C9A—C10A	117.6 (3)	C20B—C21B—C22B	121.0 (3)
C18A—C9A—N1A	119.9 (3)	C22B—C21B—H21B	119.5
C18A—C9A—C10A	122.4 (3)	C21B—C22B—C23B	121.9 (3)
C9A—C10A—H10A	120.7	C21B—C22B—C27B	119.3 (3)
C11A—C10A—C9A	118.7 (3)	C27B—C22B—C23B	118.8 (3)
C11A—C10A—H10A	120.7	C22B—C23B—H23B	119.6
C10A—C11A—H11A	119.4	C24B—C23B—C22B	120.8 (4)
C10A—C11A—C12A	121.2 (3)	C24B—C23B—H23B	119.6
C12A—C11A—H11A	119.4	C23B—C24B—H24B	120.0
C13A—C12A—C11A	121.9 (3)	C23B—C24B—C25B	119.9 (4)
C17A—C12A—C11A	119.1 (3)	C25B—C24B—H24B	120.0
C17A—C12A—C13A	119.0 (3)	C24B—C25B—H25B	119.7
C12A—C13A—H13A	119.7	C26B—C25B—C24B	120.7 (4)
C14A—C13A—C12A	120.6 (3)	C26B—C25B—H25B	119.7
C14A—C13A—H13A	119.7	C25B—C26B—H26B	119.7
C13A—C14A—H14A	119.7	C25B—C26B—C27B	120.7 (4)
C13A—C14A—C15A	120.7 (3)	C27B—C26B—H26B	119.7
C15A—C14A—H14A	119.7	C22B—C27B—C28B	119.3 (3)
C14A—C15A—H15A	120.1	C26B—C27B—C22B	119.1 (3)
C16A—C15A—C14A	119.9 (3)	C26B—C27B—C28B	121.6 (3)
C16A—C15A—H15A	120.1	C19B—C28B—C27B	119.4 (3)
C15A—C16A—H16A	119.5	C19B—C28B—H28B	120.3
C15A—C16A—C17A	120.9 (3)	C27B—C28B—H28B	120.3
C17A—C16A—H16A	119.5	N3B—C29B—C30B	119.4 (3)
C12A—C17A—C16A	118.9 (3)	C38B—C29B—N3B	119.0 (3)
C18A—C17A—C12A	119.3 (3)	C38B—C29B—C30B	121.6 (3)
C18A—C17A—C16A	121.8 (3)	C29B—C30B—H30B	120.3
C9A—C18A—C17A	119.2 (3)	C31B—C30B—C29B	119.3 (3)
C9A—C18A—H18A	120.4	C31B—C30B—H30B	120.3
C17A—C18A—H18A	120.4	C30B—C31B—H31B	119.6
N2A—C19A—C20A	118.8 (3)	C30B—C31B—C32B	120.8 (3)
C28A—C19A—N2A	119.2 (3)	C32B—C31B—H31B	119.6
C28A—C19A—C20A	122.0 (3)	C31B—C32B—C33B	121.6 (3)
C19A—C20A—H20A	120.5	C31B—C32B—C37B	119.5 (3)
C21A—C20A—C19A	119.1 (3)	C33B—C32B—C37B	118.8 (3)
C21A—C20A—H20A	120.5	C32B—C33B—H33B	119.9
C20A—C21A—H21A	119.6	C34B—C33B—C32B	120.2 (4)
C20A—C21A—C22A	120.8 (3)	C34B—C33B—H33B	119.9
C22A—C21A—H21A	119.6	C33B—C34B—H34B	119.7
C21A—C22A—C23A	121.7 (3)	C33B—C34B—C35B	120.7 (3)
C27A—C22A—C21A	119.4 (3)	C35B—C34B—H34B	119.7
C27A—C22A—C23A	118.9 (3)	C34B—C35B—H35B	119.6
C22A—C23A—H23A	119.8	C36B—C35B—C34B	120.8 (3)
C24A—C23A—C22A	120.4 (3)	C36B—C35B—H35B	119.6
C24A—C23A—H23A	119.8	C35B—C36B—H36B	120.0
C23A—C24A—H24A	119.7	C35B—C36B—C37B	120.1 (4)
C23A—C24A—C25A	120.5 (3)	C37B—C36B—H36B	120.0
C25A—C24A—H24A	119.7	C36B—C37B—C32B	119.4 (3)
C24A—C25A—H25A	119.5	C36B—C37B—C38B	121.7 (4)
C26A—C25A—C24A	120.9 (3)	C38B—C37B—C32B	118.9 (3)
C26A—C25A—H25A	119.5	C29B—C38B—C37B	119.8 (3)
C25A—C26A—H26A	120.2	C29B—C38B—H38B	120.1
C25A—C26A—C27A	119.7 (4)	C37B—C38B—H38B	120.1
C27A—C26A—H26A	120.2	C40B—C39B—C5B	122.9 (3)
C22A—C27A—C26A	119.6 (3)	C44B—C39B—C5B	119.6 (3)
C22A—C27A—C28A	119.3 (3)	C44B—C39B—C40B	117.5 (3)
C28A—C27A—C26A	121.1 (3)	C39B—C40B—H40B	119.6
C19A—C28A—C27A	119.4 (3)	C41B—C40B—C39B	120.8 (3)
C19A—C28A—H28A	120.3	C41B—C40B—H40B	119.6
C27A—C28A—H28A	120.3	C40B—C41B—H41B	119.7
N3A—C29A—C30A	118.5 (3)	C42B—C41B—C40B	120.6 (3)
C38A—C29A—N3A	119.7 (3)	C42B—C41B—H41B	119.7
C38A—C29A—C30A	121.7 (3)	C41B—C42B—H42B	120.2
C29A—C30A—H30A	120.6	C41B—C42B—C43B	119.7 (3)
C31A—C30A—C29A	118.7 (3)	C43B—C42B—H42B	120.2
C31A—C30A—H30A	120.6	C42B—C43B—H43B	120.1
C30A—C31A—H31A	119.4	C42B—C43B—C44B	119.8 (3)
C30A—C31A—C32A	121.2 (3)	C44B—C43B—H43B	120.1
C32A—C31A—H31A	119.4	C39B—C44B—H44B	119.2
C31A—C32A—C33A	122.2 (3)	C43B—C44B—C39B	121.6 (3)
C31A—C32A—C37A	119.3 (3)	C43B—C44B—H44B	119.2
C37A—C32A—C33A	118.6 (3)	C46B—C45B—C6B	124.6 (3)
C32A—C33A—H33A	119.7	C46B—C45B—C50B	117.7 (3)
C34A—C33A—C32A	120.7 (4)	C50B—C45B—C6B	117.5 (3)
C34A—C33A—H33A	119.7	C45B—C46B—H46B	119.6
C33A—C34A—H34A	119.8	C45B—C46B—C47B	120.9 (3)
C33A—C34A—C35A	120.4 (3)	C47B—C46B—H46B	119.6
C35A—C34A—H34A	119.8	C46B—C47B—H47B	119.7
C34A—C35A—H35A	119.8	C48B—C47B—C46B	120.6 (3)
C36A—C35A—C34A	120.4 (4)	C48B—C47B—H47B	119.7
C36A—C35A—H35A	119.8	C47B—C48B—H48B	120.4
C35A—C36A—H36A	119.4	C49B—C48B—C47B	119.3 (3)
C35A—C36A—C37A	121.2 (4)	C49B—C48B—H48B	120.4
C37A—C36A—H36A	119.4	C48B—C49B—H49B	119.8
C36A—C37A—C32A	118.8 (3)	C48B—C49B—C50B	120.4 (3)
C38A—C37A—C32A	118.8 (3)	C50B—C49B—H49B	119.8
C38A—C37A—C36A	122.4 (3)	C45B—C50B—H50B	119.4
C29A—C38A—C37A	120.3 (3)	C49B—C50B—C45B	121.2 (3)
C29A—C38A—H38A	119.8	C49B—C50B—H50B	119.4
C37A—C38A—H38A	119.8	C52B—C51B—C7B	119.5 (3)
C40A—C39A—C5A	122.4 (3)	C56B—C51B—C7B	121.7 (3)
C40A—C39A—C44A	117.8 (3)	C56B—C51B—C52B	118.5 (3)
C44A—C39A—C5A	119.8 (3)	C51B—C52B—H52B	119.9
C39A—C40A—H40A	119.5	C53B—C52B—C51B	120.2 (3)
C41A—C40A—C39A	121.0 (3)	C53B—C52B—H52B	119.9
C41A—C40A—H40A	119.5	C52B—C53B—H53B	119.8
C40A—C41A—H41A	119.8	C54B—C53B—C52B	120.4 (3)
C42A—C41A—C40A	120.4 (3)	C54B—C53B—H53B	119.8
C42A—C41A—H41A	119.8	C53B—C54B—H54B	120.0
C41A—C42A—H42A	120.5	C53B—C54B—C55B	119.9 (3)
C43A—C42A—C41A	119.0 (3)	C55B—C54B—H54B	120.0
C43A—C42A—H42A	120.5	C54B—C55B—H55B	120.2
C42A—C43A—H43A	119.6	C56B—C55B—C54B	119.5 (3)
C42A—C43A—C44A	120.8 (3)	C56B—C55B—H55B	120.2
C44A—C43A—H43A	119.6	C51B—C56B—H56B	119.3
C39A—C44A—H44A	119.5	C55B—C56B—C51B	121.4 (3)
C43A—C44A—C39A	121.0 (3)	C55B—C56B—H56B	119.3
C43A—C44A—H44A	119.5	C58B—C57B—C8B	119.5 (3)
C46A—C45A—C6A	124.1 (3)	C58B—C57B—C62B	118.3 (3)
C46A—C45A—C50A	118.6 (3)	C62B—C57B—C8B	121.9 (3)
C50A—C45A—C6A	117.3 (3)	C57B—C58B—H58B	119.7
C45A—C46A—H46A	119.6	C59B—C58B—C57B	120.6 (3)
C47A—C46A—C45A	120.9 (3)	C59B—C58B—H58B	119.7
C47A—C46A—H46A	119.6	C58B—C59B—H59B	119.6
C46A—C47A—H47A	120.1	C58B—C59B—C60B	120.8 (3)
C48A—C47A—C46A	119.7 (3)	C60B—C59B—H59B	119.6
C48A—C47A—H47A	120.1	C59B—C60B—H60B	120.4
C47A—C48A—H48A	120.0	C59B—C60B—C61B	119.2 (3)
C49A—C48A—C47A	120.0 (3)	C61B—C60B—H60B	120.4
C49A—C48A—H48A	120.0	C60B—C61B—H61B	120.0
C48A—C49A—H49A	119.8	C62B—C61B—C60B	119.9 (3)
C48A—C49A—C50A	120.3 (3)	C62B—C61B—H61B	120.0
C50A—C49A—H49A	119.8	C57B—C62B—H62B	119.4
C45A—C50A—H50A	119.8	C61B—C62B—C57B	121.1 (3)
C49A—C50A—C45A	120.4 (3)	C61B—C62B—H62B	119.4
C49A—C50A—H50A	119.8	O1—C1—C2	121.2 (3)
C52A—C51A—C7A	118.0 (3)	O1—C1—C3	122.7 (3)
C56A—C51A—C7A	123.5 (2)	C3—C1—C2	116.1 (3)
C56A—C51A—C52A	118.4 (3)	C1—C2—H2A	109.5
C51A—C52A—H52A	119.6	C1—C2—H2B	109.5
C51A—C52A—C53A	120.7 (3)	C1—C2—H2C	109.5
C53A—C52A—H52A	119.6	H2A—C2—H2B	109.5
C52A—C53A—H53A	119.9	H2A—C2—H2C	109.5
C54A—C53A—C52A	120.2 (3)	H2B—C2—H2C	109.5
C54A—C53A—H53A	119.9	C1—C3—H3A	109.5
C53A—C54A—H54A	120.2	C1—C3—H3B	109.5
C55A—C54A—C53A	119.6 (3)	C1—C3—H3C	109.5
C55A—C54A—H54A	120.2	H3A—C3—H3B	109.5
C54A—C55A—H55A	119.7	H3A—C3—H3C	109.5
C54A—C55A—C56A	120.5 (3)	H3B—C3—H3C	109.5
C56A—C55A—H55A	119.7	N3B—C29C—C38C	108 (2)
C51A—C56A—C55A	120.5 (3)	N3B—C29C—C30C	127 (2)
C51A—C56A—H56A	119.7	C38C—C29C—C30C	124 (2)
C55A—C56A—H56A	119.7	C29C—C38C—H38C	121.8
C58A—C57A—C8A	119.5 (3)	C29C—C38C—C37C	116 (3)
C62A—C57A—C8A	122.4 (3)	C37C—C38C—H38C	121.8
C62A—C57A—C58A	118.0 (3)	C36C—C37C—C38C	120 (2)
C57A—C58A—H58A	119.7	C36C—C37C—C32C	118 (2)
C59A—C58A—C57A	120.6 (3)	C32C—C37C—C38C	121 (2)
C59A—C58A—H58A	119.7	C37C—C36C—H36C	120.0
C58A—C59A—H59A	119.7	C37C—C36C—C35C	120 (3)
C60A—C59A—C58A	120.7 (3)	C35C—C36C—H36C	120.0
C60A—C59A—H59A	119.7	C36C—C35C—H35C	120.6
C59A—C60A—H60A	120.4	C36C—C35C—C34C	119 (2)
C59A—C60A—C61A	119.2 (3)	C34C—C35C—H35C	120.6
C61A—C60A—H60A	120.4	C35C—C34C—H34C	117.8
C60A—C61A—H61A	119.7	C33C—C34C—C35C	124 (3)
C62A—C61A—C60A	120.6 (3)	C33C—C34C—H34C	117.8
C62A—C61A—H61A	119.7	C34C—C33C—H33C	123.1
C57A—C62A—H62A	119.5	C34C—C33C—C32C	114 (3)
C61A—C62A—C57A	120.9 (3)	C32C—C33C—H33C	123.1
C61A—C62A—H62A	119.5	C37C—C32C—C33C	124 (2)
C1B—Fe1B—C2B	98.74 (12)	C37C—C32C—C31C	120 (2)
C1B—Fe1B—C4B	78.86 (11)	C31C—C32C—C33C	116 (2)
C1B—Fe1B—C5B	98.60 (12)	C32C—C31C—H31C	120.4
C1B—Fe1B—C6B	138.56 (12)	C30C—C31C—C32C	119 (2)
C1B—Fe1B—C7B	134.61 (12)	C30C—C31C—H31C	120.4
C1B—Fe1B—C8B	94.92 (12)	C29C—C30C—C31C	119 (2)
C2B—Fe1B—C4B	138.73 (11)	C29C—C30C—H30C	120.3
C2B—Fe1B—C5B	160.37 (12)	C31C—C30C—H30C	120.3
C2B—Fe1B—C6B	121.14 (12)	C20C—C19C—N2B	126 (2)
C2B—Fe1B—C7B	93.89 (12)	C20C—C19C—C28C	126 (2)
C2B—Fe1B—C8B	102.83 (12)	C28C—C19C—N2B	107.0 (19)
C3B—Fe1B—C1B	92.42 (13)	C26C—C27C—C22C	117 (2)
C3B—Fe1B—C2B	95.40 (13)	C26C—C27C—C28C	119 (2)
C3B—Fe1B—C4B	125.77 (11)	C28C—C27C—C22C	124 (2)
C3B—Fe1B—C5B	92.98 (12)	C24C—C25C—H25C	121.8
C3B—Fe1B—C6B	94.38 (12)	C24C—C25C—C26C	116 (3)
C3B—Fe1B—C7B	129.62 (12)	C26C—C25C—H25C	121.8
C3B—Fe1B—C8B	159.03 (11)	C19C—C20C—H20C	122.7
C5B—Fe1B—C4B	37.99 (10)	C19C—C20C—C21C	115 (2)
C5B—Fe1B—C8B	66.52 (11)	C21C—C20C—H20C	122.7
C6B—Fe1B—C4B	64.17 (11)	C25C—C24C—H24C	118.0
C6B—Fe1B—C5B	40.28 (11)	C25C—C24C—C23C	124 (3)
C6B—Fe1B—C7B	40.59 (10)	C23C—C24C—H24C	118.0
C6B—Fe1B—C8B	67.38 (11)	C20C—C21C—H21C	117.5
C7B—Fe1B—C4B	63.59 (10)	C20C—C21C—C22C	125 (2)
C7B—Fe1B—C5B	67.24 (11)	C22C—C21C—H21C	117.5
C7B—Fe1B—C8B	39.70 (11)	C27C—C26C—H26C	118.2
C8B—Fe1B—C4B	37.75 (10)	C25C—C26C—C27C	124 (3)
C1B—N1B—C9B	162.6 (3)	C25C—C26C—H26C	118.2
C2B—N2B—C19B	175.8 (3)	C21C—C22C—C27C	115 (2)
C2B—N2B—C19C	157.9 (12)	C23C—C22C—C27C	123 (3)
C3B—N3B—C29B	165.0 (3)	C23C—C22C—C21C	122 (2)
C3B—N3B—C29C	152.5 (14)	C19C—C28C—C27C	114 (2)
N1B—C1B—Fe1B	173.4 (2)	C19C—C28C—H28C	122.8
N2B—C2B—Fe1B	178.2 (3)	C27C—C28C—H28C	122.8
N3B—C3B—Fe1B	175.0 (3)	C24C—C23C—C22C	117 (3)
O1B—C4B—Fe1B	135.8 (2)	C24C—C23C—H23C	121.7
O1B—C4B—C5B	127.7 (3)	C22C—C23C—H23C	121.7
O1B—C4B—C8B	127.9 (3)		
			
Fe1A—C4A—C5A—C6A	−61.44 (18)	O1B—C4B—C8B—C7B	−169.1 (3)
Fe1A—C4A—C5A—C39A	132.8 (3)	O1B—C4B—C8B—C57B	−5.2 (5)
Fe1A—C4A—C8A—C7A	61.26 (17)	N1B—C9B—C10B—C11B	−178.6 (3)
Fe1A—C4A—C8A—C57A	−132.7 (3)	N1B—C9B—C18B—C17B	178.2 (3)
Fe1A—C5A—C6A—C7A	−60.74 (17)	N2B—C19B—C20B—C21B	179.5 (3)
Fe1A—C5A—C6A—C45A	130.8 (3)	N2B—C19B—C28B—C27B	179.0 (3)
Fe1A—C5A—C39A—C40A	−55.7 (4)	N2B—C19C—C20C—C21C	169 (3)
Fe1A—C5A—C39A—C44A	127.9 (3)	N2B—C19C—C28C—C27C	−170.8 (19)
Fe1A—C6A—C7A—C8A	−63.09 (18)	N3B—C29B—C30B—C31B	−178.1 (3)
Fe1A—C6A—C7A—C51A	129.6 (3)	N3B—C29B—C38B—C37B	178.4 (3)
Fe1A—C6A—C45A—C46A	−26.3 (4)	N3B—C29C—C38C—C37C	−167 (2)
Fe1A—C6A—C45A—C50A	154.1 (2)	N3B—C29C—C30C—C31C	167 (3)
Fe1A—C7A—C8A—C4A	−69.26 (19)	C1B—N1B—C9B—C10B	156.2 (9)
Fe1A—C7A—C8A—C57A	124.9 (3)	C1B—N1B—C9B—C18B	−22.3 (11)
Fe1A—C7A—C51A—C52A	−151.0 (2)	C2B—N2B—C19C—C20C	14 (6)
Fe1A—C7A—C51A—C56A	32.5 (4)	C2B—N2B—C19C—C28C	−176 (2)
Fe1A—C8A—C57A—C58A	−132.2 (3)	C3B—N3B—C29B—C30B	131.6 (11)
Fe1A—C8A—C57A—C62A	50.9 (4)	C3B—N3B—C29B—C38B	−48.0 (14)
O1A—C4A—C5A—Fe1A	−126.0 (3)	C3B—N3B—C29C—C38C	161 (2)
O1A—C4A—C5A—C6A	172.5 (3)	C3B—N3B—C29C—C30C	−5 (6)
O1A—C4A—C5A—C39A	6.8 (5)	C4B—C5B—C6B—Fe1B	71.13 (19)
O1A—C4A—C8A—Fe1A	126.1 (3)	C4B—C5B—C6B—C7B	9.7 (3)
O1A—C4A—C8A—C7A	−172.7 (3)	C4B—C5B—C6B—C45B	−157.8 (3)
O1A—C4A—C8A—C57A	−6.6 (5)	C4B—C5B—C39B—C40B	135.0 (3)
N1A—C9A—C10A—C11A	−177.3 (3)	C4B—C5B—C39B—C44B	−45.2 (4)
N1A—C9A—C18A—C17A	177.3 (2)	C4B—C8B—C57B—C58B	−41.2 (4)
N2A—C19A—C20A—C21A	−177.0 (3)	C4B—C8B—C57B—C62B	144.8 (3)
N2A—C19A—C28A—C27A	175.9 (3)	C5B—C4B—C8B—Fe1B	−46.74 (18)
N3A—C29A—C30A—C31A	−177.0 (3)	C5B—C4B—C8B—C7B	15.8 (3)
N3A—C29A—C38A—C37A	176.8 (3)	C5B—C4B—C8B—C57B	179.7 (3)
C1A—N1A—C9A—C10A	−3.5 (16)	C5B—C6B—C7B—Fe1B	62.24 (18)
C1A—N1A—C9A—C18A	177.4 (13)	C5B—C6B—C7B—C8B	0.4 (3)
C3A—N3A—C29A—C30A	102.3 (15)	C5B—C6B—C7B—C51B	−170.2 (2)
C3A—N3A—C29A—C38A	−75.6 (16)	C5B—C6B—C45B—C46B	−85.4 (4)
C4A—C5A—C6A—Fe1A	69.26 (19)	C5B—C6B—C45B—C50B	99.1 (4)
C4A—C5A—C6A—C7A	8.5 (3)	C5B—C39B—C40B—C41B	177.9 (3)
C4A—C5A—C6A—C45A	−160.0 (2)	C5B—C39B—C44B—C43B	−178.3 (3)
C4A—C5A—C39A—C40A	−162.5 (3)	C6B—C5B—C39B—C40B	−33.2 (5)
C4A—C5A—C39A—C44A	21.1 (4)	C6B—C5B—C39B—C44B	146.6 (3)
C4A—C8A—C57A—C58A	−25.2 (4)	C6B—C7B—C8B—Fe1B	59.85 (18)
C4A—C8A—C57A—C62A	157.9 (3)	C6B—C7B—C8B—C4B	−10.4 (3)
C5A—C4A—C8A—Fe1A	−48.41 (18)	C6B—C7B—C8B—C57B	−174.4 (3)
C5A—C4A—C8A—C7A	12.9 (3)	C6B—C7B—C51B—C52B	126.9 (3)
C5A—C4A—C8A—C57A	178.9 (2)	C6B—C7B—C51B—C56B	−46.1 (4)
C5A—C6A—C7A—Fe1A	62.81 (18)	C6B—C45B—C46B—C47B	−175.1 (3)
C5A—C6A—C7A—C8A	−0.3 (3)	C6B—C45B—C50B—C49B	175.8 (3)
C5A—C6A—C7A—C51A	−167.6 (2)	C7B—C6B—C45B—C46B	108.7 (3)
C5A—C6A—C45A—C46A	−125.5 (3)	C7B—C6B—C45B—C50B	−66.8 (4)
C5A—C6A—C45A—C50A	54.9 (4)	C7B—C8B—C57B—C58B	120.2 (3)
C5A—C39A—C40A—C41A	−175.7 (3)	C7B—C8B—C57B—C62B	−53.8 (4)
C5A—C39A—C44A—C43A	176.0 (3)	C7B—C51B—C52B—C53B	−172.7 (3)
C6A—C5A—C39A—C40A	34.1 (4)	C7B—C51B—C56B—C55B	170.3 (3)
C6A—C5A—C39A—C44A	−142.3 (3)	C8B—C4B—C5B—Fe1B	47.35 (19)
C6A—C7A—C8A—Fe1A	61.18 (17)	C8B—C4B—C5B—C6B	−15.6 (3)
C6A—C7A—C8A—C4A	−8.1 (3)	C8B—C4B—C5B—C39B	174.2 (2)
C6A—C7A—C8A—C57A	−173.9 (2)	C8B—C7B—C51B—C52B	−41.9 (4)
C6A—C7A—C51A—C52A	114.8 (3)	C8B—C7B—C51B—C56B	145.1 (3)
C6A—C7A—C51A—C56A	−61.7 (4)	C8B—C57B—C58B—C59B	−171.8 (3)
C6A—C45A—C46A—C47A	−178.4 (3)	C8B—C57B—C62B—C61B	172.4 (3)
C6A—C45A—C50A—C49A	179.3 (3)	C9B—C10B—C11B—C12B	0.1 (4)
C7A—C6A—C45A—C46A	67.9 (4)	C10B—C9B—C18B—C17B	−0.3 (4)
C7A—C6A—C45A—C50A	−111.7 (3)	C10B—C11B—C12B—C13B	−180.0 (3)
C7A—C8A—C57A—C58A	138.5 (3)	C10B—C11B—C12B—C17B	0.4 (4)
C7A—C8A—C57A—C62A	−38.4 (4)	C11B—C12B—C13B—C14B	−177.4 (3)
C7A—C51A—C52A—C53A	−174.2 (3)	C11B—C12B—C17B—C16B	177.3 (3)
C7A—C51A—C56A—C55A	173.3 (3)	C11B—C12B—C17B—C18B	−0.8 (4)
C8A—C4A—C5A—Fe1A	48.41 (18)	C12B—C13B—C14B—C15B	−0.8 (5)
C8A—C4A—C5A—C6A	−13.0 (3)	C12B—C17B—C18B—C9B	0.8 (4)
C8A—C4A—C5A—C39A	−178.8 (2)	C13B—C12B—C17B—C16B	−2.3 (4)
C8A—C7A—C51A—C52A	−50.1 (4)	C13B—C12B—C17B—C18B	179.5 (3)
C8A—C7A—C51A—C56A	133.4 (3)	C13B—C14B—C15B—C16B	−0.7 (5)
C8A—C57A—C58A—C59A	−176.7 (3)	C14B—C15B—C16B—C17B	0.6 (5)
C8A—C57A—C62A—C61A	176.1 (3)	C15B—C16B—C17B—C12B	0.9 (5)
C9A—C10A—C11A—C12A	0.6 (5)	C15B—C16B—C17B—C18B	179.1 (3)
C10A—C9A—C18A—C17A	−1.7 (4)	C16B—C17B—C18B—C9B	−177.3 (3)
C10A—C11A—C12A—C13A	176.4 (3)	C17B—C12B—C13B—C14B	2.3 (5)
C10A—C11A—C12A—C17A	−2.9 (4)	C18B—C9B—C10B—C11B	−0.2 (4)
C11A—C12A—C13A—C14A	−179.4 (3)	C19B—C20B—C21B—C22B	1.5 (5)
C11A—C12A—C17A—C16A	−178.9 (3)	C20B—C19B—C28B—C27B	0.5 (5)
C11A—C12A—C17A—C18A	3.0 (4)	C20B—C21B—C22B—C23B	178.9 (3)
C12A—C13A—C14A—C15A	−1.1 (5)	C20B—C21B—C22B—C27B	0.4 (5)
C12A—C17A—C18A—C9A	−0.7 (4)	C21B—C22B—C23B—C24B	−177.3 (4)
C13A—C12A—C17A—C16A	1.8 (4)	C21B—C22B—C27B—C26B	177.3 (4)
C13A—C12A—C17A—C18A	−176.4 (3)	C21B—C22B—C27B—C28B	−1.9 (5)
C13A—C14A—C15A—C16A	0.7 (5)	C22B—C23B—C24B—C25B	−0.3 (6)
C14A—C15A—C16A—C17A	1.1 (5)	C22B—C27B—C28B—C19B	1.4 (6)
C15A—C16A—C17A—C12A	−2.3 (4)	C23B—C22B—C27B—C26B	−1.3 (6)
C15A—C16A—C17A—C18A	175.8 (3)	C23B—C22B—C27B—C28B	179.6 (3)
C16A—C17A—C18A—C9A	−178.8 (3)	C23B—C24B—C25B—C26B	−0.7 (6)
C17A—C12A—C13A—C14A	−0.1 (5)	C24B—C25B—C26B—C27B	0.7 (6)
C18A—C9A—C10A—C11A	1.8 (4)	C25B—C26B—C27B—C22B	0.3 (6)
C19A—C20A—C21A—C22A	0.9 (5)	C25B—C26B—C27B—C28B	179.5 (4)
C20A—C19A—C28A—C27A	−2.3 (5)	C26B—C27B—C28B—C19B	−177.7 (4)
C20A—C21A—C22A—C23A	177.3 (3)	C27B—C22B—C23B—C24B	1.2 (5)
C20A—C21A—C22A—C27A	−1.7 (5)	C28B—C19B—C20B—C21B	−2.0 (5)
C21A—C22A—C23A—C24A	−178.8 (3)	C29B—C30B—C31B—C32B	−0.4 (5)
C21A—C22A—C27A—C26A	179.0 (3)	C30B—C29B—C38B—C37B	−1.1 (5)
C21A—C22A—C27A—C28A	0.5 (4)	C30B—C31B—C32B—C33B	179.5 (3)
C22A—C23A—C24A—C25A	−0.3 (6)	C30B—C31B—C32B—C37B	−1.0 (5)
C22A—C27A—C28A—C19A	1.5 (4)	C31B—C32B—C33B—C34B	178.9 (3)
C23A—C22A—C27A—C26A	0.0 (5)	C31B—C32B—C37B—C36B	−179.1 (3)
C23A—C22A—C27A—C28A	−178.5 (3)	C31B—C32B—C37B—C38B	1.3 (5)
C23A—C24A—C25A—C26A	0.2 (7)	C32B—C33B—C34B—C35B	0.5 (6)
C24A—C25A—C26A—C27A	−0.1 (6)	C32B—C37B—C38B—C29B	−0.3 (5)
C25A—C26A—C27A—C22A	−0.1 (5)	C33B—C32B—C37B—C36B	0.4 (5)
C25A—C26A—C27A—C28A	178.4 (4)	C33B—C32B—C37B—C38B	−179.1 (3)
C26A—C27A—C28A—C19A	−177.1 (3)	C33B—C34B—C35B—C36B	−0.2 (6)
C27A—C22A—C23A—C24A	0.1 (5)	C34B—C35B—C36B—C37B	−0.1 (6)
C28A—C19A—C20A—C21A	1.1 (5)	C35B—C36B—C37B—C32B	−0.1 (5)
C29A—C30A—C31A—C32A	−0.7 (5)	C35B—C36B—C37B—C38B	179.5 (3)
C30A—C29A—C38A—C37A	−1.0 (5)	C36B—C37B—C38B—C29B	−179.8 (3)
C30A—C31A—C32A—C33A	−179.8 (3)	C37B—C32B—C33B—C34B	−0.7 (5)
C30A—C31A—C32A—C37A	0.8 (5)	C38B—C29B—C30B—C31B	1.5 (5)
C31A—C32A—C33A—C34A	−178.3 (3)	C39B—C5B—C6B—Fe1B	−119.3 (3)
C31A—C32A—C37A—C36A	178.6 (3)	C39B—C5B—C6B—C7B	179.3 (3)
C31A—C32A—C37A—C38A	−0.9 (4)	C39B—C5B—C6B—C45B	11.7 (5)
C32A—C33A—C34A—C35A	−0.5 (6)	C39B—C40B—C41B—C42B	1.5 (6)
C32A—C37A—C38A—C29A	1.0 (4)	C40B—C39B—C44B—C43B	1.5 (5)
C33A—C32A—C37A—C36A	−0.9 (5)	C40B—C41B—C42B—C43B	−0.7 (7)
C33A—C32A—C37A—C38A	179.6 (3)	C41B—C42B—C43B—C44B	0.3 (6)
C33A—C34A—C35A—C36A	−0.5 (6)	C42B—C43B—C44B—C39B	−0.8 (6)
C34A—C35A—C36A—C37A	0.8 (6)	C44B—C39B—C40B—C41B	−1.9 (5)
C35A—C36A—C37A—C32A	−0.1 (5)	C45B—C6B—C7B—Fe1B	−129.3 (3)
C35A—C36A—C37A—C38A	179.4 (3)	C45B—C6B—C7B—C8B	168.9 (2)
C36A—C37A—C38A—C29A	−178.5 (3)	C45B—C6B—C7B—C51B	−1.7 (4)
C37A—C32A—C33A—C34A	1.2 (5)	C45B—C46B—C47B—C48B	0.1 (5)
C38A—C29A—C30A—C31A	0.8 (5)	C46B—C45B—C50B—C49B	0.0 (5)
C39A—C5A—C6A—Fe1A	−125.3 (3)	C46B—C47B—C48B—C49B	−0.9 (5)
C39A—C5A—C6A—C7A	174.0 (2)	C47B—C48B—C49B—C50B	1.3 (5)
C39A—C5A—C6A—C45A	5.5 (4)	C48B—C49B—C50B—C45B	−0.9 (5)
C39A—C40A—C41A—C42A	−0.5 (5)	C50B—C45B—C46B—C47B	0.4 (4)
C40A—C39A—C44A—C43A	−0.6 (4)	C51B—C7B—C8B—Fe1B	−130.0 (3)
C40A—C41A—C42A—C43A	0.0 (5)	C51B—C7B—C8B—C4B	159.7 (3)
C41A—C42A—C43A—C44A	0.2 (5)	C51B—C7B—C8B—C57B	−4.3 (4)
C42A—C43A—C44A—C39A	0.1 (5)	C51B—C52B—C53B—C54B	1.9 (5)
C44A—C39A—C40A—C41A	0.8 (4)	C52B—C51B—C56B—C55B	−2.8 (4)
C45A—C6A—C7A—Fe1A	−128.7 (3)	C52B—C53B—C54B—C55B	−2.1 (5)
C45A—C6A—C7A—C8A	168.2 (2)	C53B—C54B—C55B—C56B	−0.1 (5)
C45A—C6A—C7A—C51A	0.9 (4)	C54B—C55B—C56B—C51B	2.6 (5)
C45A—C46A—C47A—C48A	−1.0 (5)	C56B—C51B—C52B—C53B	0.6 (4)
C46A—C45A—C50A—C49A	−0.3 (4)	C57B—C58B—C59B—C60B	−1.3 (5)
C46A—C47A—C48A—C49A	0.0 (5)	C58B—C57B—C62B—C61B	−1.7 (4)
C47A—C48A—C49A—C50A	0.8 (5)	C58B—C59B—C60B—C61B	−0.7 (5)
C48A—C49A—C50A—C45A	−0.7 (5)	C59B—C60B—C61B—C62B	1.5 (5)
C50A—C45A—C46A—C47A	1.2 (4)	C60B—C61B—C62B—C57B	−0.3 (5)
C51A—C7A—C8A—Fe1A	−131.8 (3)	C62B—C57B—C58B—C59B	2.5 (5)
C51A—C7A—C8A—C4A	158.9 (3)	C29C—C38C—C37C—C36C	179 (2)
C51A—C7A—C8A—C57A	−6.9 (4)	C29C—C38C—C37C—C32C	1 (2)
C51A—C52A—C53A—C54A	−0.3 (5)	C38C—C29C—C30C—C31C	4 (4)
C52A—C51A—C56A—C55A	−3.2 (4)	C38C—C37C—C36C—C35C	−180 (2)
C52A—C53A—C54A—C55A	−1.2 (5)	C38C—C37C—C32C—C33C	−178 (3)
C53A—C54A—C55A—C56A	0.5 (5)	C38C—C37C—C32C—C31C	−2 (4)
C54A—C55A—C56A—C51A	1.7 (5)	C37C—C36C—C35C—C34C	2 (4)
C56A—C51A—C52A—C53A	2.5 (4)	C37C—C32C—C31C—C30C	4 (5)
C57A—C58A—C59A—C60A	0.3 (5)	C36C—C37C—C32C—C33C	3 (5)
C58A—C57A—C62A—C61A	−0.8 (4)	C36C—C37C—C32C—C31C	179 (3)
C58A—C59A—C60A—C61A	−0.5 (5)	C36C—C35C—C34C—C33C	−5 (5)
C59A—C60A—C61A—C62A	0.1 (5)	C35C—C34C—C33C—C32C	7 (5)
C60A—C61A—C62A—C57A	0.6 (5)	C34C—C33C—C32C—C37C	−6 (5)
C62A—C57A—C58A—C59A	0.4 (4)	C34C—C33C—C32C—C31C	178 (3)
Fe1B—C4B—C5B—C6B	−62.95 (18)	C33C—C32C—C31C—C30C	−180 (3)
Fe1B—C4B—C5B—C39B	126.9 (3)	C32C—C37C—C36C—C35C	−1 (4)
Fe1B—C4B—C8B—C7B	62.52 (18)	C32C—C31C—C30C—C29C	−5 (5)
Fe1B—C4B—C8B—C57B	−133.5 (3)	C30C—C29C—C38C—C37C	−1 (3)
Fe1B—C5B—C6B—C7B	−61.40 (18)	C19C—C20C—C21C—C22C	−1 (4)
Fe1B—C5B—C6B—C45B	131.1 (3)	C27C—C22C—C23C—C24C	3 (5)
Fe1B—C5B—C39B—C40B	−122.1 (3)	C25C—C24C—C23C—C22C	−3 (4)
Fe1B—C5B—C39B—C44B	57.8 (4)	C20C—C19C—C28C—C27C	−1 (4)
Fe1B—C6B—C7B—C8B	−61.83 (18)	C20C—C21C—C22C—C27C	1 (4)
Fe1B—C6B—C7B—C51B	127.5 (3)	C20C—C21C—C22C—C23C	−178 (3)
Fe1B—C6B—C45B—C46B	16.0 (4)	C24C—C25C—C26C—C27C	1 (3)
Fe1B—C6B—C45B—C50B	−159.4 (2)	C21C—C22C—C23C—C24C	−178 (3)
Fe1B—C7B—C8B—C4B	−70.24 (19)	C26C—C27C—C22C—C21C	180 (2)
Fe1B—C7B—C8B—C57B	125.7 (3)	C26C—C27C—C22C—C23C	−1 (4)
Fe1B—C7B—C51B—C52B	−142.0 (2)	C26C—C27C—C28C—C19C	180 (2)
Fe1B—C7B—C51B—C56B	44.9 (4)	C26C—C25C—C24C—C23C	1 (2)
Fe1B—C8B—C57B—C58B	−149.9 (2)	C22C—C27C—C26C—C25C	−1 (4)
Fe1B—C8B—C57B—C62B	36.1 (4)	C22C—C27C—C28C—C19C	1 (4)
O1B—C4B—C5B—Fe1B	−127.7 (3)	C28C—C19C—C20C—C21C	0 (4)
O1B—C4B—C5B—C6B	169.3 (3)	C28C—C27C—C26C—C25C	180 (2)
O1B—C4B—C5B—C39B	−0.8 (5)	C28C—C27C—C22C—C21C	−1 (4)
O1B—C4B—C8B—Fe1B	128.3 (3)	C28C—C27C—C22C—C23C	178 (3)
